# Paraoxonase 2 (PON2) plays a limited role in murine lung tumorigenesis

**DOI:** 10.1038/s41598-023-37146-5

**Published:** 2023-06-19

**Authors:** Aaron G. Whitt, Aaron M. Neely, Omar Sadi Sarkar, Shuhan Meng, Sengodagounder Arumugam, Kavitha Yaddanapudi, Chi Li

**Affiliations:** 1 Department of Pharmacology and Toxicology, University of Louisville, Louisville, KY 40202 USA; 2 Experimental Therapeutics Group, Brown Cancer Center, Department of Medicine, University of Louisville, Louisville, KY 40202 USA; 3 Department of Microbiology and Immunology, University of Louisville, Louisville, KY USA; 4 NMR Facility, Brown Cancer Center, Department of Medicine, University of Louisville, Louisville, KY USA; 5 Immuno-Oncology Program, Brown Cancer Center, Department of Medicine, University of Louisville, Louisville, KY USA; 6 Division of Immunotherapy, Department of Surgery, University of Louisville, Louisville, KY USA; 7 Present Address: Department of Translational Genomics, University of Southern California, Los Angeles, CA USA

**Keywords:** Cancer, Cell biology

## Abstract

Paraoxonase 2 (PON2) is a multifunctional intracellular enzyme that has received growing attention for its ability to modulate various aspects of normal and malignant cellular physiology. Recent research has revealed that PON2 is upregulated in tissues from patients with various types of solid tumors and hematologic cancers, likely due to its ability to suppress oxidative stress and evade apoptosis. However, the effects of PON2 on pulmonary oncogenesis are unknown. Here, we conducted studies to investigate how PON2 influences lung cancer cell proliferation in vitro and lung tumorigenesis in vivo using a variety of cellular and animal models. It was found that PON2 expression deficiency hampered the proliferation of cultured lung cancer cells with concomitant cell cycle arrest at the G1 phase. In addition, the loss of endogenous PON2 expression impaired key aspects of oxidative metabolism in lung adenocarcinoma cells. Moreover, we investigated how the interplay between PON2 expression in lung tumors and host mice influences lung tumor initiation and progression. PON2 status in both transplanted tumor cells and mice failed to influence the development of subcutaneously grafted Lewis lung carcinoma (LLC) tumors, orthotopically implanted LLC tumors, and oncogenic Kras-driven primary lung adenocarcinoma tumors. Importantly, the frequencies of tumor-infiltrating myeloid subsets that include myeloid-derived suppressor cells (MDSCs) and tumor-associated macrophages were not impacted by PON2 expression in LLC tumor-bearing mice. Overall, our studies indicate that PON2 plays a limited role in murine lung tumorigenesis.

## Introduction

Paraoxonase 2 (PON2) is an intracellular lactonase/arylesterase enzyme with antioxidant properties^1–3^. The current body of research into PON2 biology has shown that neoplastic cells utilize PON2 activity to counteract oxidative stress and escape apoptosis^4–7^. Consistent with these observations, PON2 is upregulated in various solid tumors and hematologic cancers^8,9^. A limited but growing number of studies have also demonstrated that PON2 expression negatively correlates with cancer patient prognoses. In a cohort of pediatric and adult patients with B cell acute lymphoblastic leukemia (B-ALL), high PON2 mRNA expression was associated with reduced overall survival and relapse-free survival^10^. In patients with cutaneous melanoma and basal cell carcinoma, higher expression of PON2 protein was correlated with important prognostic indicators, including Breslow thickness, Clark level, regression, lymph node metastases, and pathological stage^11^. Similarly, Wang et al. reported that elevated PON2 expression in patients with gastric cancer was unfavorably associated with clinical stage, invasion, lymph node metastasis, distant metastasis, and overall patient survival^12^. Furthermore, the prognostic value of PON2 expression was also explored in patients with bladder cancer, which revealed that PON2 was upregulated in early-stage tumors and fell in later-stage tumors, suggesting a potentially important role for PON2 in different stages of tumorigenesis^13^.

Recent research efforts have implemented murine models of tumorigenesis to explore PON2’s influence on malignant growth in vivo with diverse results. In two murine models of B-ALL driven by the oncogenic fusion protein BRC-ABL1 or constitutively active NRAS^G12D^, the loss of PON2 expression impaired disease progression and drastically improved survival duration in transplant recipient mice^10^. Researchers also investigated PON2’s role in pancreatic ductal adenocarcinoma (PDAC) tumor growth and metastasis using multiple murine tumor models^14^. This report demonstrates that PON2 expression is necessary for subcutaneously and orthotopically implanted xenografts of the PDAC cell line PANC1. Furthermore, a decrease in PON2 expression reduced PDAC metastases to liver (from splenic injection) and lung (from tail vein injection) compared with PDAC cells with normal PON2 expression. Conversely, a tumor suppressor role for PON2 was identified in the context of ovarian cancer^15^. In this study, elevated PON2 expression blunted ovarian cancer cell proliferation and xenograft tumor growth in mice through inhibiting insulin-like growth factor-1 (IGF-1) expression and signaling transduction^15^. These reported discrepancies suggest PON2’s influence on tumor growth in vivo likely depends on microenvironmental factors corresponding to distinct organs and tissues that give rise to neoplastic growth.

Several studies within the past decade have reported that PON2 is upregulated in lung tumors^8^, including lung adenocarcinoma^9,16^. However, the effects of PON2 on lung cancer initiation and progression are unknown. Therefore, we conducted studies to investigate how PON2 influences lung cancer cell proliferation in vitro and lung tumorigenesis in vivo using a variety of cellular and animal models. These studies were performed in immunocompetent mice of both sexes to further address the potential interplay of PON2 expression and host factors, such as macrophage polarization, in mediating pulmonary oncogenesis.

## Results

### PON2 promotes the proliferation of murine lung carcinoma cells

**Figure 1:**
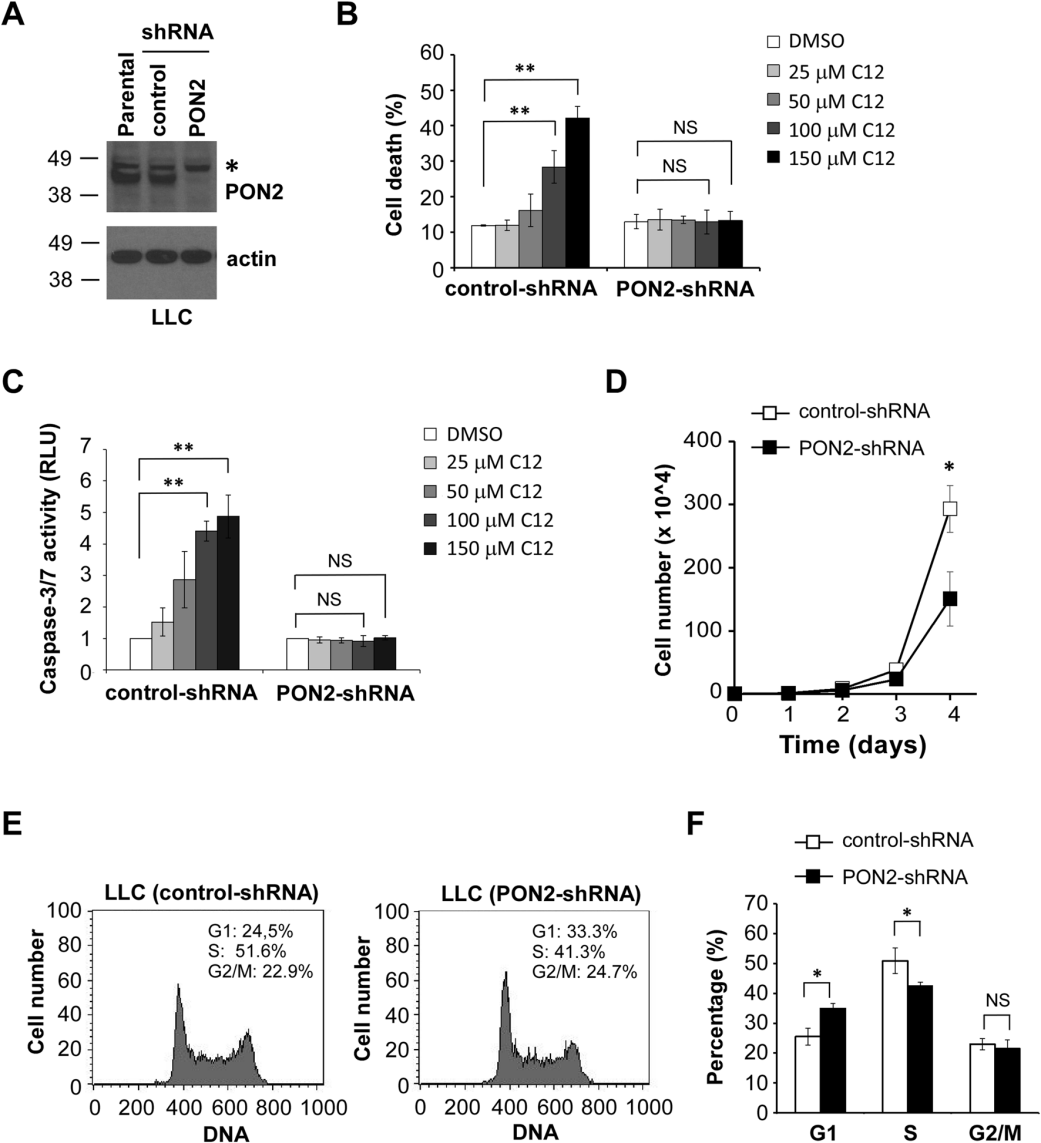
Reducing PON2 expression impairs the proliferation of murine cancer cell LLC. (**A**) PON2 expression in the indicated LLC cells was detected by western blot analysis. The molecular weight markers are labeled on the left (kD). “*” indicates a non-specific band. (**B**) Control- and PON2-shRNA LLC cells were treated with increasing concentrations of C12 for 24 h and cell viability was determined by PI exclusion via flow cytometry. Data are mean ± SD of three independent experiments. (**C**) Caspase-3/7 activation in the indicated LLC cells was measured following 2-h exposure to DMSO or C12. Mean ± SD of three independent experiments is shown. (**D**) Cell proliferation was measured by cell counting over a four-day period in control- and PON2-shRNA LLC cells. Data are presented as mean ± SD of three independent experiments. (**E**) DNA content in control- and PON2-shRNA LLC cells was measured by flow cytometry. (**F**) Data shown in (E) were analyzed using FloJo software to determine the cell cycle stages of LLC cells. Mean ± SD of three independent experiments is shown. For all the data, asterisks indicate *p*-values of ˂ 0.05 (*), ˂ 0.01 (**) by Student’s unpaired t test. *NS* No significance.

To explore PON2’s role in pulmonary oncogenesis, we first sought to investigate how PON2 may impact cellular proliferation of murine Lewis lung carcinoma (LLC) cells. To this purpose, PON2 expression in LLC cells was stably reduced by RNAi (Fig. 1A). To further validate the reduction of PON2 expression, we conducted cell viability and caspase-3/7 activity assays in control- and PON2-shRNA LLC cells treated with increasing concentrations of the bacterial quorum-sensing molecule *N*-(3-oxododecanoyl)-l-homoserine lactone (C12), a specific inducer of PON2-mediated apoptosis^16–19^. While control-shRNA LLC cells exhibited a concentration-dependent increase in cell death and caspase-3/7 activation upon C12 exposure, cells lacking PON2 expression were resistant to the cytotoxicity of C12 at all concentrations (Fig. 1B,C), in agreement with earlier studies of various cell types^16–19^. Next, we examined cellular proliferation in LLCs with or without PON2 expression. As shown in Fig. 1D. the decrease of PON2 expression impaired LLC proliferation compared with their counterparts expressing control-shRNA, demonstrating an important role for PON2 to promote cell proliferation in murine lung carcinoma cells. To further explore the consequence of reduced PON2 expression on cell cycle progression, we evaluated the DNA content of LLC cells with normal or reduced PON2 (Fig. 1E). LLC cells deficient in PON2 expression displayed a higher percentage of cells in G1 phase and a lower proportion of cells in S phase compared with their control counterparts (Fig. 1F), indicating that PON2 plays an important role for the proliferation of cultured murine lung tumor cells.

### PON2 is required for human lung adenocarcinoma cell proliferation but not nontransformed cell proliferation

**Figure 2:**
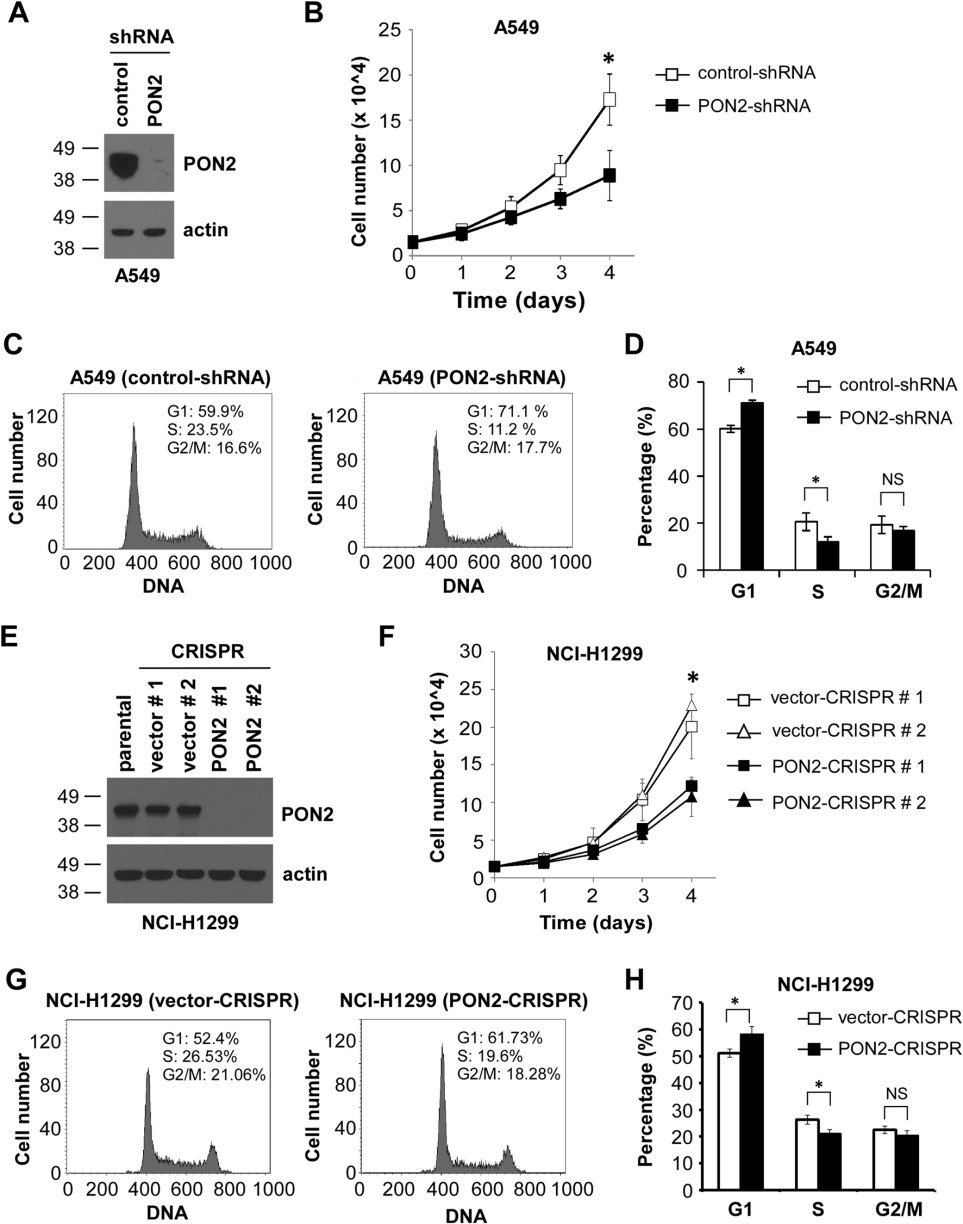
PON2 is required for human lung adenocarcinoma cellular proliferation. (**A**) PON2 expression was reduced in A549 cells by RNAi. Western blot analysis shows PON2 protein levels in A549 cells expressing control- or PON2-specific shRNA. The molecular weight markers are labeled on the left (kD). (**B**) The proliferation of the indicated A549 cells was determined by cell counting. Data are presented as mean ± SD of three independent experiments. (**C**) Representative cell cycle profiles of the indicated A549 cells are shown. (**D**) The cell cycle stages of A549 cells shown in (**C**) were evaluated. Data are presented as mean ± SD of three independent experiments. (**E**) PON2 expression was analyzed by western blot in the indicated NCI-H1299 cell lines. The molecular weight markers are labeled on the left (kD). (**F**) The proliferation of NCI-H1299 cells was determined by cell counting. Mean ± SD of three independent experiments is shown. (**G**) The cell cycle profile of NCI-H1299 vector-CRISPR (line #1) and PON2-CRISPR (line #1) cells was evaluated. (**H**) The cell cycle stages of NCI-H1299 cells presented in (G) were examined. Data are mean ± SD of three independent experiments. For all the data, asterisks indicate *p*-values of < 0.05 (*), < 0.01 (**) by Student’s unpaired t-test. *NS* No significance.

Since PON2 expression has been found to increase in human lung adenocarcinoma^9,16^, we further investigated the role of PON2 in cellular proliferation of two human lung adenocarcinoma cell lines, A549 and NCI-H1299 cells. As before, we employed an RNAi approach to reduce PON2 expression in A549 cells (Fig. 2A), and the loss of PON2 expression resulted in impaired proliferation of A549 cells (Fig. 2B). Furthermore, cell cycle profile studies demonstrated that A549 cells deficient in PON2 expression were more likely to arrest at the G1 phase compared with their control counterparts (Fig. 2C,D). For NCI-H1299 cells, we utilized a CRISPR/Cas9 approach to disrupt endogenous PON2 expression, and western blot analysis confirmed the elimination of PON2 expression in PON2-CRISPR NCI-H1299 clonal lines (Fig. 2E). Similar to LLC and A549 cells (Figs. 1D and 2B), the lack of PON2 expression in NCI-H1299 cells caused a decrease in cell growth (Fig. 2F), further validating PON2’s role in regulating lung cancer cell proliferation. Again, the loss of PON2 expression arrested NCI-H1299 cell cycle progression at the G1 phase (Fig. 2G,H). Taken together, these results illustrate an important role for PON2 to promote cell proliferation in oncogenically transformed pulmonary epithelial cells.

To explore the involvement of PON2 in the proliferation of nontransformed cells, endogenous PON2 expression was stably reduced in human embryonic kidney-293T (HEK-293T) cells and immortalized human bronchial epithelial (HBE) cells by RNAi (Supplementary Fig. 1A,D). Reducing PON2 expression in HEK-293 cells and HBE cells conferred resistance to C12 (Supplementary Fig. 1B,E). HEK-293T and HBE cells lacking PON2 expression proliferated at the same rate as their counterparts expressing the empty vector (Supplementary Fig. 1C,F), indicating that PON2 expression does not govern the proliferation of nontransformed cells. Together, the loss of PON2 expression specifically reduced the proliferation of oncogenically transformed lung epithelial cells, highlighting PON2 as a potentially selective target against lung cancer cell growth.

### The loss of PON2 disrupts cellular metabolism in lung adenocarcinoma cells

**Figure 3:**
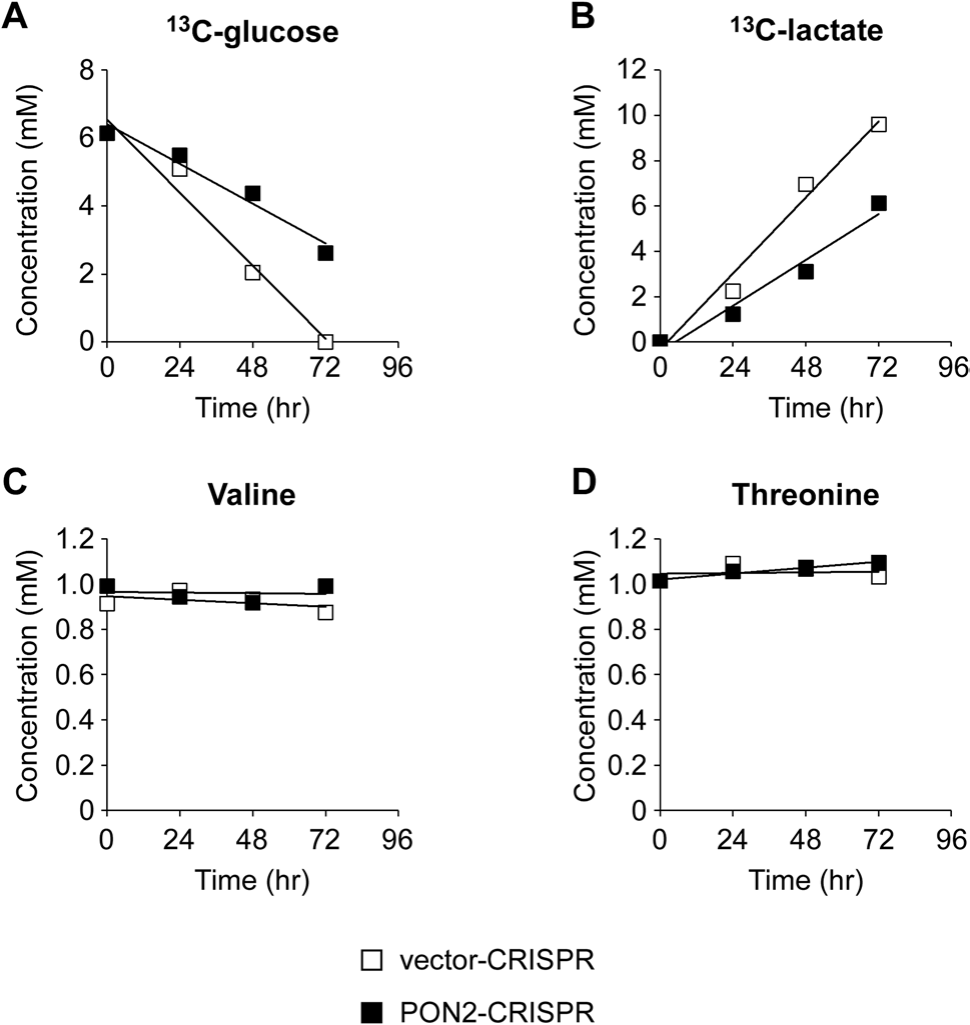
PON2 deficiency impairs extracellular glucose consumption and lactate production in NCI-H1299 cells. Vector- and PON2-CRISPR NCI-H1299 cells were cultured in the medium supplemented with [U-^13^C]-glucose. The culture medium was collected at 0-, 24-, 48- and 72-h timepoints. Key extracellular metabolites, including ^13^C-glucose (**A**), ^13^C-lactate (**B**), valine (**C**), and threonine (**D**) were quantified by 1D ^1^H NMR analysis. Lines are linear regression fits.

Given that loss of PON2 expression was detrimental to lung adenocarcinoma cell proliferation (Fig. 2), we sought to investigate the impact of PON2 on cellular bioenergetics through a stable isotope resolved metabolomics (SIRM) approach. Using high-resolution nuclear magnetic resonance (NMR), we tracked the flow of [U-^13^C]-glucose through oxidative metabolic networks to explore PON2’s influence on key metrics of cellular metabolism, including glycolysis, the tricarboxylic acid (TCA) cycle, pyrimidine biosynthesis, and pentose phosphate pathway (PPP) activity (Supplementary Fig. 2). We first evaluated the abundance of key extracellular metabolites in the growth medium of NCI-H1299 cells cultured in the presence of [U-^13^C]-glucose using one dimensional (1D) ^1^H NMR analysis. At time 0 h, the peaks corresponding to ^13^C-glucose, valine, and threonine were detected in the culture medium (Supplementary Fig. 3A). Analysis of the extracellular medium following 72-h incubation revealed different metabolite profiles corresponding to PON2 status (Supplementary Fig. 3B,C). The peak corresponding to ^13^C-glucose was detected in the 72-h sample prepared from PON2-CRISPR, but not vector-CRISPR cells, showing that PON2 promotes the consumption of extracellular glucose. Furthermore, the abundance of ^13^C-lactate was higher in vector-CRISPR samples at the 72-h timepoint, indicating that the loss of PON2 expression impairs lactate secretion. The essential amino acids valine and threonine were detected at similar abundances regardless of PON2 status. Metabolite abundances over time were plotted for samples and a linear regression was applied to examine the rate of metabolite depletion and production (Fig. 3). In agreement with the data acquired at 72-h timepoint, the loss of PON2 expression reduced the rates of ^13^C-glucose consumption (Fig. 3A) and ^13^C-lactate (Fig. 3B) production, while valine and threonine concentrations were unaltered (Fig. 3C,D).

To obtain detailed insight into PON2’s role in oxidative metabolism within lung adenocarcinoma cells, we analyzed the abundances of labeled intracellular metabolites produced from [U-^13^C]-glucose using two dimensional (2D) TOCSY analysis. The abundances of ^13^C-labeled metabolites represent de novo biosynthesis originated from glucose as a carbon source and are reflective of glycolysis, the TCA cycle, PPP activity, and nucleotide biosynthesis reactions. As shown in Supplementary Fig. 4, alanine displayed the characteristic square pattern of ^13^C satellites surrounding a central resonance that represents the unlabeled molecule, demonstrating that alanine was synthesized directly from glucose through glycolysis^20,21^. In PON2-deficient NCI-H1299 cells, we found a decreased abundance of ^13^C-alanine (Table 1), suggesting that glycolytic activity was compromised in PON2-CRISPR cells.

**Table 1:** Anabolic activities of mitochondria in cells lacking PON2 expression are compromised.

Molecule	Site	Vector-CRISPR	PON2-CRISPR
Alanine	^12^C2^12^C3	33.3	49.9
	^13^C2^13^C3	66.7	50.1
Glutamate	^12^C2^12^C4	43.8	55.8
	^12^C2^13^C4	6.6	4.8
	^13^C2^12^C4	32.8	27.6
	^13^C2^13^C4	16.9	11.8
Glutamine	^12^C2^12^C4	46.4	63.7
	^12^C2^13^C4	3.5	3.7
	^13^C2^12^C4	30.6	19.6
	^13^C2^13^C4	19.5	13.0
Aspartate	^12^C2^12^C3	39.2	64.2
	^12^C2^13^C3	24.6	20.8
	^13^C2^12^C3	14.4	15.0
	^13^C2^13^C3	21.8	0
Uracil in UTP	^12^C6^12^C5	52.5	62.5
	^12^C6^13^C5	17.2	12.7
	^13^C6^12^C5	15.2	12.6
	^13^C6^13^C5	15.2	12.1
UTP	^12^C1^12^C2	11.7	6.5
	^13^C2^13^C2	88.3	93.5

The indicated metabolites in NCI-H1299 vector-CRISPR and PON2-CRISPR cells were calculated by integration, with correction for saturation where necessary. ^13^C enrichment was quantified using the peak satellites as demonstrated by TOCSY (see Supplementary Fig. 4). ^13^C-labeling in cellular alanine, glutamate, glutamine, aspartate, and uracil in UTP was reduced in CRISPR-PON2 cells compared with their vector control counterparts.

To assess TCA cycle activity, the relative abundances of labeled glutamate, glutamine, and aspartate were also assessed. For glutamate and glutamine, we detected a complex labeling pattern in which 4 separate species of each were present: unlabeled, ^13^C labeling at both positions (C2 and C4), and ^13^C at only one position (C2 or C4) (Supplementary Fig. 4A). While unlabeled glutamate/glutamine are present in culture medium, labeled species are produced from α-ketoglutarate, an intermediate metabolite in the TCA cycle, via transaminases (α-ketoglutarate → glutamate) and glutamine synthetase (glutamate → glutamine) (Supplementary Fig. 2). The degree of ^13^C-labeling present in α-ketoglutarate dictates whether one or both positions in glutamate/glutamine possess ^13^C atoms. Similarly, ^13^C-labeled species of aspartate are derived from the TCA cycle intermediate oxaloacetate (Supplementary Fig. 2). This conversion occurs in mitochondria via the activity of aspartate aminotransferase as part of the malate-aspartate shuttle. PON2-CRISPR cells had reduced relative abundances of ^13^C-labeled glutamate, glutamine, and aspartate species compared with vector-CRISPR cells, suggesting PON2 deficiency impairs TCA cycle activity (Table 1), consistent with the decreased glucose uptake and flow through glycolysis (Fig. 3).

The results of this experimental approach also provided insight into PON2’s role in de novo pyrimidine nucleotide biosynthesis by monitoring the incorporation of ^13^C atoms in uracil in UTP. In proliferating tumor cells, the existing pool of nucleotides is insufficient and must be synthesized de novo. In mitochondria, oxaloacetate can be converted to aspartate and eventually to uracil base (Supplementary Fig. 2). Therefore, examining the labeling patterns of uracil in UTP is an effective surrogate for de novo pyrimidine biosynthesis. In this SIRM analysis, we found that the ^13^C labeling of uracil in UTP was decreased in PON2-CRISPR cells (Table 1), which might be due to reduced TCA cycle activity.

Finally, we explored the role of PON2 in PPP activity, via the labeling patterns of UTP-ribose (Supplementary Fig. 4B). PON2-CRISPR cells had a higher relative abundance of ^13^C-labeled UTP-ribose (Table 1), although the difference was slight. This indicates that PPP activity is elevated following loss of PON2 expression. Since PPP activity is involved in the production of reducing equivalents, such as NADPH, and nucleotide precursors, it is conceivable that cells deficient in PON2 expression maintain PPP activity to support cellular proliferation and redox status within cells. Taken together, the results of SIRM studies demonstrate that the loss of endogenous PON2 expression impairs key aspects of oxidative metabolism in lung adenocarcinoma cells.

### PON2 plays a limited role in the development of subcutaneously implanted lung tumors

**Figure 4:**
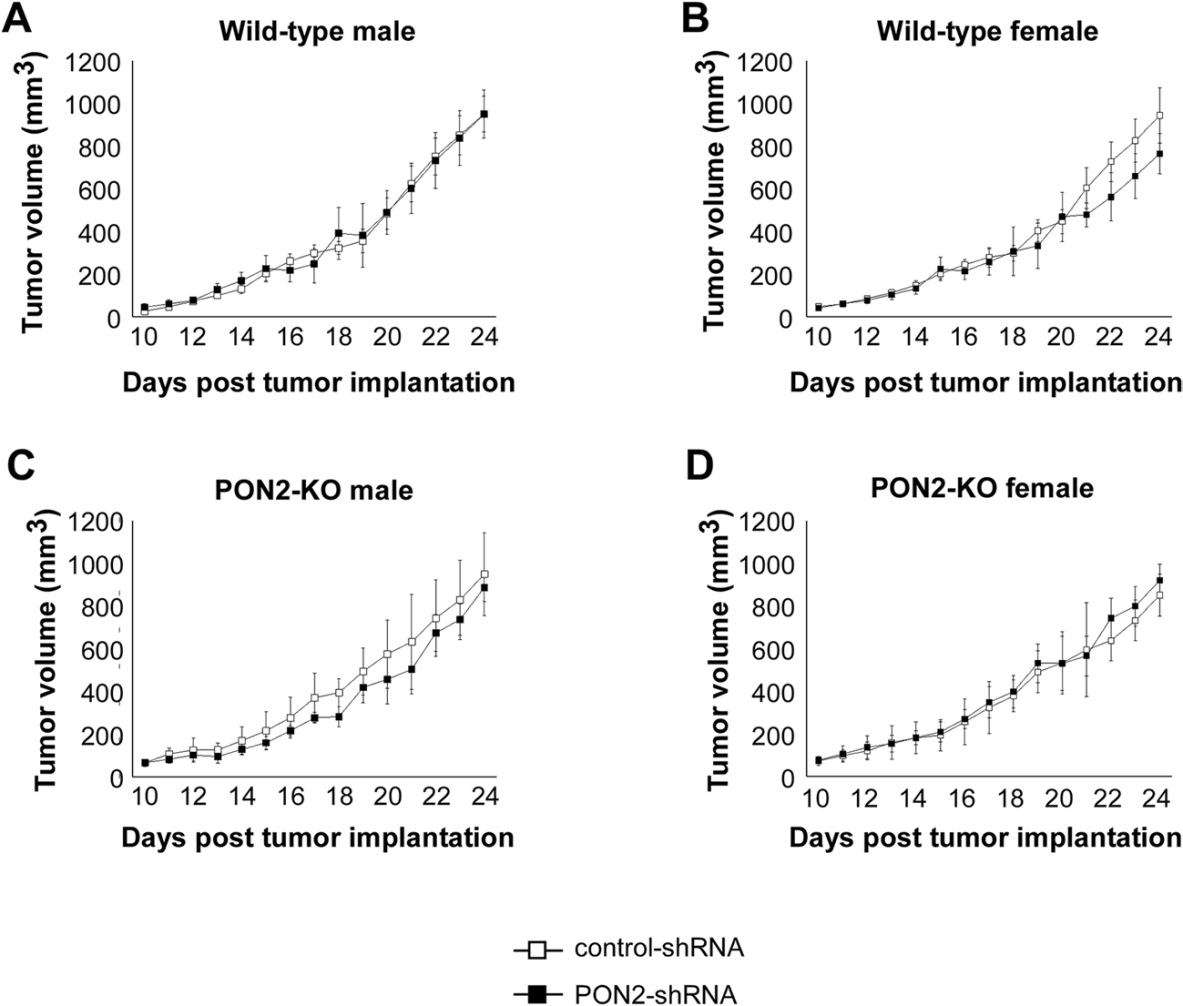
The growth of subcutaneously grafted LLC tumors is independent of PON2 expression levels. LLC cells expressing control-shRNA or PON2-shRNA (1 × 10^5^) were injected into the rear flanks of wild-type male (**A**), wild-type female (**B**), PON2-KO male (**C**), or PON2-KO female (**D**) mice, and tumor burden was examined over 24 days. Data are mean ± SD for 5 mice per group.

As PON2 expression deficiency hampered the proliferation of cultured lung tumor cells (Figs. 1 and 2), we sought to explore its role in pulmonary tumorigenesis in mice. Recently, a study demonstrated that PON2 promoted an M2 anti-inflammatory phenotype in murine peritoneal macrophages^22^. Since M2-polarized macrophages can facilitate tumor progression^23^, we reasoned that mice lacking PON2 expression would be protected from grafted tumor growth through enhanced phagocytosis of implanted tumor cells. Therefore, we investigated how the interplay between PON2 expression in transplanted lung tumors and host mice influences lung cancer progression. Furthermore, we sought to examine the impact of host mouse sex in modulating lung tumorigenesis. To this purpose, we conducted studies in a subcutaneous allograft lung tumor model in which LLC cells with or without PON2 expression were subcutaneously implanted in the rear flanks of wild-type and PON2-KO mice of both sexes, and subcutaneous tumor volume was measured over 24 days. In wild-type animals, subcutaneous tumor growth was similar between control- and PON2-shRNA groups (Fig. 4A,B). A similar pattern was discovered in mice lacking PON2 expression, in that the rate of tumor progression was unaffected by the PON2 status of grafted LLC cells (Fig. 4C,D). Furthermore, tumor growth in wild-type vs. PON2-KO animals was uniform, indicating that PON2 host status does not influence the subcutaneous growth of LLC cells. Finally, no differences were detected in tumor development between male and female mice. These results are not consistent with the finding that the loss of PON2 expression hindered the proliferation of lung cancer cell lines in vitro (Figs. 1 and 2).

### Orthotopically implanted lung tumor growth is unaffected by PON2 expression

**Figure 5:**
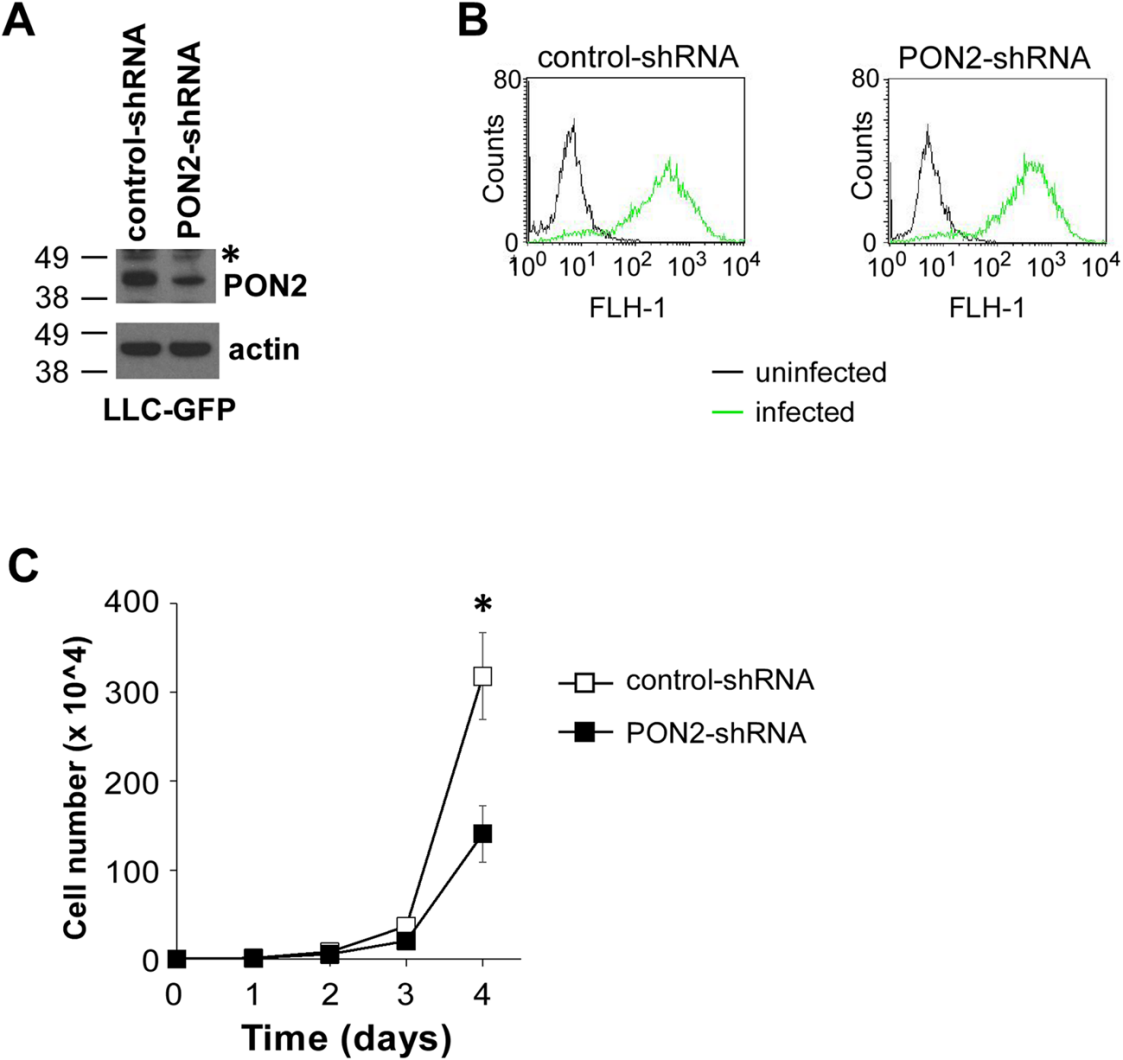
PON2 expression is required for the proliferation of murine lung carcinoma cells expressing GFP. (**A**) GFP protein was expressed in control- and PON2-shRNA LLC cells by retroviral expression, and PON2 expression was examined by western blot. The molecular weight markers are labeled on the left (kD). (**B**) Fluorescence intensity of GFP in control- and PON2-shRNA LLC cells was determined by flow cytometry. (**C**) The proliferation of GFP-positive LLC cells expressing control- or PON2-shRNA was evaluated. The data are shown as mean ± SD of three independent experiments. Asterisks indicate *p*-values of ˂ 0.05 (*) by Student’s unpaired t test.

It is notable that subcutaneous allograft lung tumor model has some deficiencies, as subcutaneous injection does not represent appropriate sites for human lung tumors^24,25^. Thus, we sought to further interrogate the influence of PON2 on lung tumor growth in an orthotopic allograft murine model of lung cancer^26^. In this approach, lung tumor cells are directly seeded into the pleural cavity, which more accurately resembles the tumor microenvironment of primary lung tumors compared with subcutaneous models of tumorigenesis. To quantify intrathoracic tumor burden, we engineered LLC cells expressing GFP protein so that GFP fluorescence could be used to distinguish tumor cells from nonmalignant pulmonary cells (Fig. 5A). Following the infection of retrovirus expressing GFP, similar GFP fluorescence intensity was detected in control- and PON2-shRNA LLC cells (Fig. 5B). To functionally validate the loss of PON2 expression, we examined cellular proliferation as before, showing that the lack of PON2 expression reduced GFP-expressing LLC cell proliferation (Fig. 5C).

**Figure 6:**
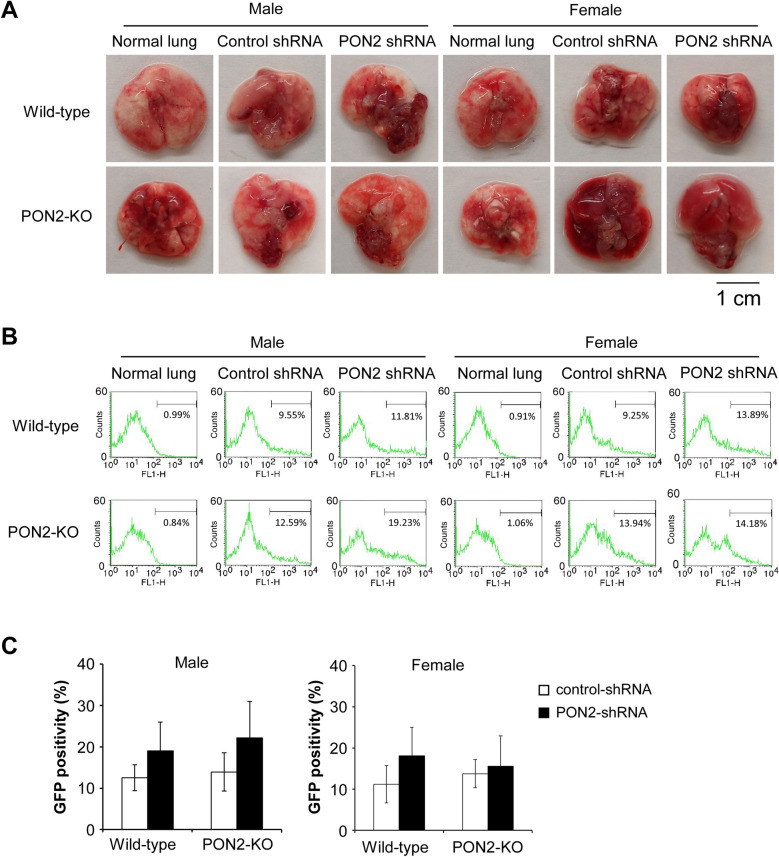
The development of intrathoracic lung tumors is not affected by PON2 expression in implanted LLC cells or host mice. (**A**) Wild-type and PON2-KO mice developed intrathoracic tumors following percutaneously administration of GFP-positive control- or PON2-shRNA LLC cells. Representative images of lung and tumor tissues acquired ten days after administering LLC cells. (**B**) Orthotopic lung tumor burden was evaluated by determining GFP-positivity of mouse lung and tumor tissues shown in (**A**). Lung tissues bearing GFP-expressing LLC tumors were collected. Single cell suspension of pulmonary cells was acquired and GFP fluorescence was evaluated by flow cytometry. Representative flow cytometry plots are shown. (**C**) Summary of the data presented in (**B**). Data are mean ± SD of 5 mice per group. Statistical significance was determined by two-way ANOVA. No significant difference was discovered as related to PON2 expression status of LLC cells, genetic background of hosts, or mouse sex.

Next, GFP-positive LLC cells with different PON2 expression levels were percutaneously injected into the thoracic cavity of wild-type and PON2-KO mice of both sexes. Tumors were allowed to develop for 10 days, at which point lung and tumor tissues were harvested (Fig. 6A). To quantify tumor burden, whole lung and tumor tissues were mechanically separated and enzymatically digested to generate a single cell suspension, and GFP positivity was evaluated by flow cytometry (Fig. 6B). Tumor burden was measured as the percentage of GFP-positive cells present in each sample according to a published protocol^27^. This experimental approach showed that intrathoracic tumor burden was similar among different experimental conditions (Fig. 6C). At experimental endpoint, tumors formed by LLCs lacking PON2 expression were similar in size to their control counterparts. Furthermore, host status, including PON2 expression levels and sex, failed to alter the progression of intrathoracic tumors. Again, there is a discrepancy between cultured lung tumor cell growth in vitro (Figs. 1 and 2) and grafted lung tumor progression in vivo (Fig. 6).

### Primary lung adenocarcinoma development in mice is independent of PON2 expression

It is known that implanted lung tumor models have some deficiencies, as subcutaneous injection or orthotopic administration of LLC cells does not recapitulate the initiation and progression processes of primary lung tumors, particularly with respect to the lack of the interaction between tumor cells and their physiological environment^24,25^. To overcome these drawbacks, we developed a primary lung adenocarcinoma mouse model to investigate the role of PON2 in pulmonary tumorigenesis. Kras is among the most frequently mutated oncogenes in lung adenocarcinoma^28,29^. An earlier study first reported the use of a genetically engineered mouse strain with an inducible Kras^G12D^ allele, which is activated by the delivery of Cre recombinase to lung tissues to drive primary lung tumor formation^30^. Research in the past decade has expanded the utility of this mouse strain through selective breeding with other genetically engineered mouse strains harboring additional oncogenes^31^. In the present study, we generated Kras^LSL-G12D^ mice with or without PON2 expression by breeding PON2-deficient mice with Kras^LSL-G12D^ mice, which yielded PON2-heterozygous mice with a mutant Kras allele. These mice were subsequently bred to generate wild-type or homozygous PON2-KO mice harboring a single copy of Kras^LSL-G12D^ (wild-type/Kras^LSL-G12D^ and PON2-KO/Kras^LSL-G12D^, respectively).

**Figure 7:**
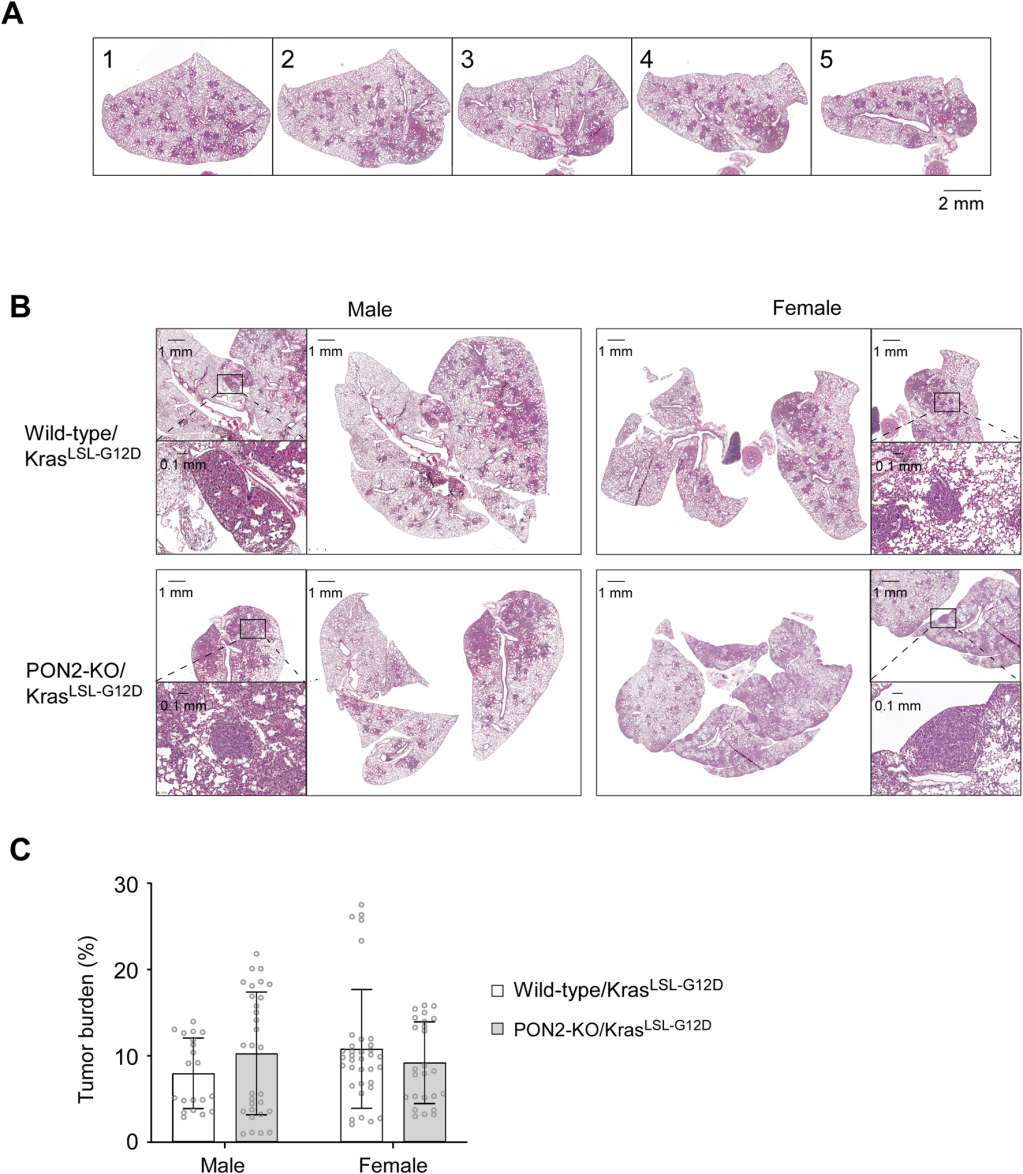
Primary lung adenocarcinoma development in mice is independent of PON2 expression. (**A**) Representative digital slide images of five serial sections of a lung separated by 200 μM and stained with hematoxalin and eosin (H&E). (**B**) Representative digital slide images of H&E-stained lungs collected from wild-type/Kras^LSL-G12D^ and PON2-KO/Kras^LSL-G12D^ mice. Inlets show selected lung tumor sections at 10X magnification. (**C**) Tumor burden (%) was quantified by comparing tumor cross-sectional area to whole lung cross-sectional area and was determined at 5 depths per lung. Male wild-type/Kras^LSL-G12D^ (n = 4 mice); male PON2-KO/Kras^LSL-G12D^ (n = 6 mice); female wild-type/Kras^LSL-G12D^ (n = 6 mice); female PON2-KO/Kras^LSL-G12D^ (n = 5 mice). Each data point in the bar graphs represents an individual tissue section. No significant differences in tumor burden were detected between wild-type and PON2-deficient mice of either sex. Data are shown as mean ± SD; each point represents an individual section. A two-way ANOVA was used to determine statistical significance.

Wild-type/Kras^LSL-G12D^ and PON2-KO/Kras^LSL-G12D^ mice of both sexes were administered with adenovirus expressing Cre recombinase (Ad-Cre) intratracheally at 6–8 weeks of age. Twenty-eight weeks following tumor induction, formalin-fixed, paraffin-embedded (FFPE) lungs were sectioned at five equidistant intervals spanning half of each lung to evaluate tumor prevalence at multiple depths (Fig. 7A). The summed cross-sectional areas of tumors were compared to total lung cross-sectional area to determine tumor burden^32^. As expected, administrating Ad-Cre was sufficient to induce the development of primary lung tumors in mice harboring the inducible mutant Kras allele (Fig. 7B). The evaluation of tumor burden for male and female mice with or without PON2 expression demonstrated that PON2 expression failed to influence the initiation and progression of oncogenic Kras-driven lung tumors (Fig. 7C). Furthermore, the tumor burden of male and female mice was comparable. These findings are similar to earlier experiments utilizing LLC cells with or without PON2 expression in subcutaneous and intrathoracic tumor models (Figs. 4 and 6) but disagree with the requirement of PON2 for lung cancer cell proliferation in vitro (Figs. 1 and 2). This discrepancy suggests an important context dependency for PON2’s role in regulating tumor cell physiology.

### The profiles of tumor-infiltrating MDSCs and macrophages are similar between host animals with or without PON2 expression

**Figure 8:**
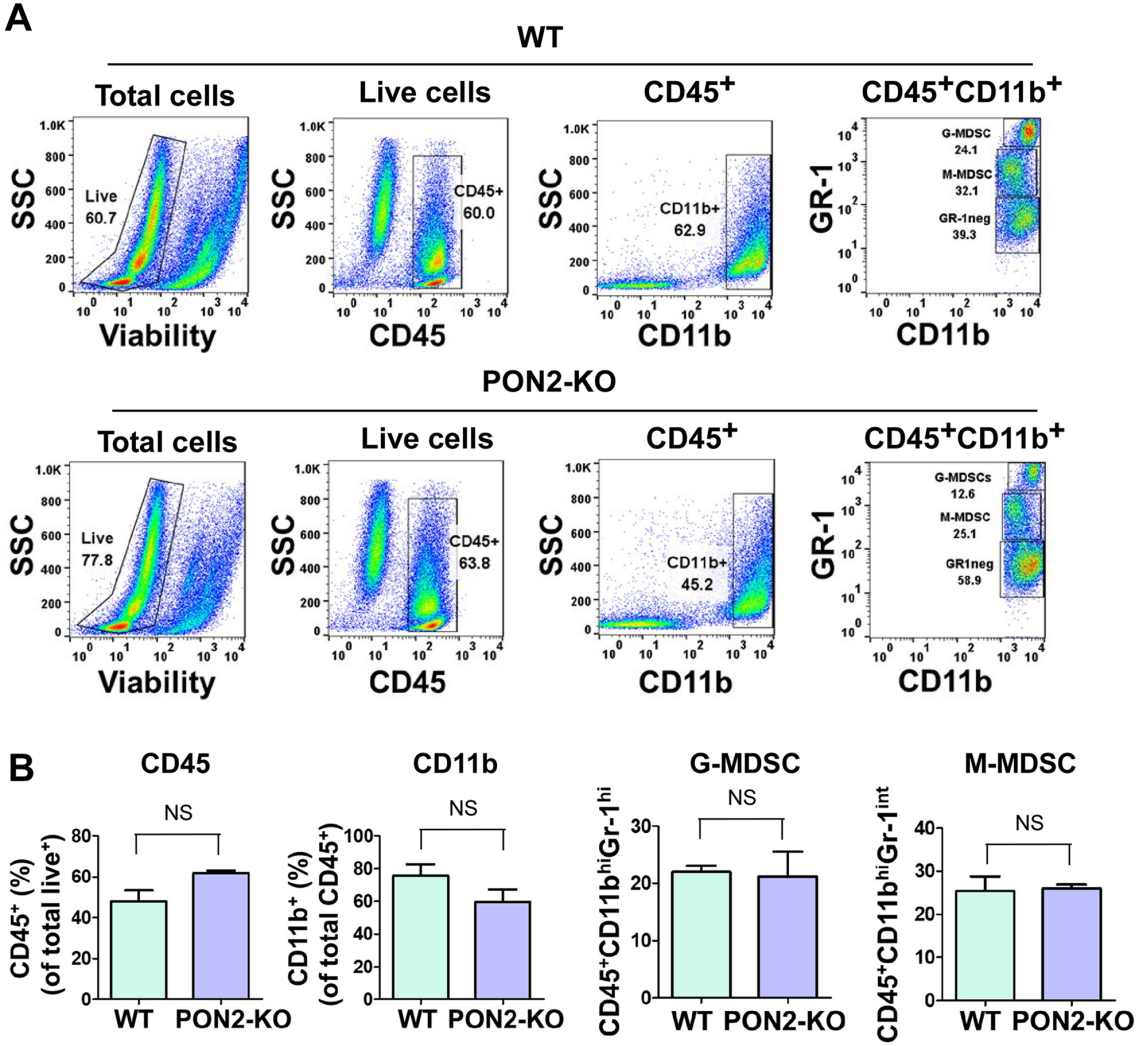

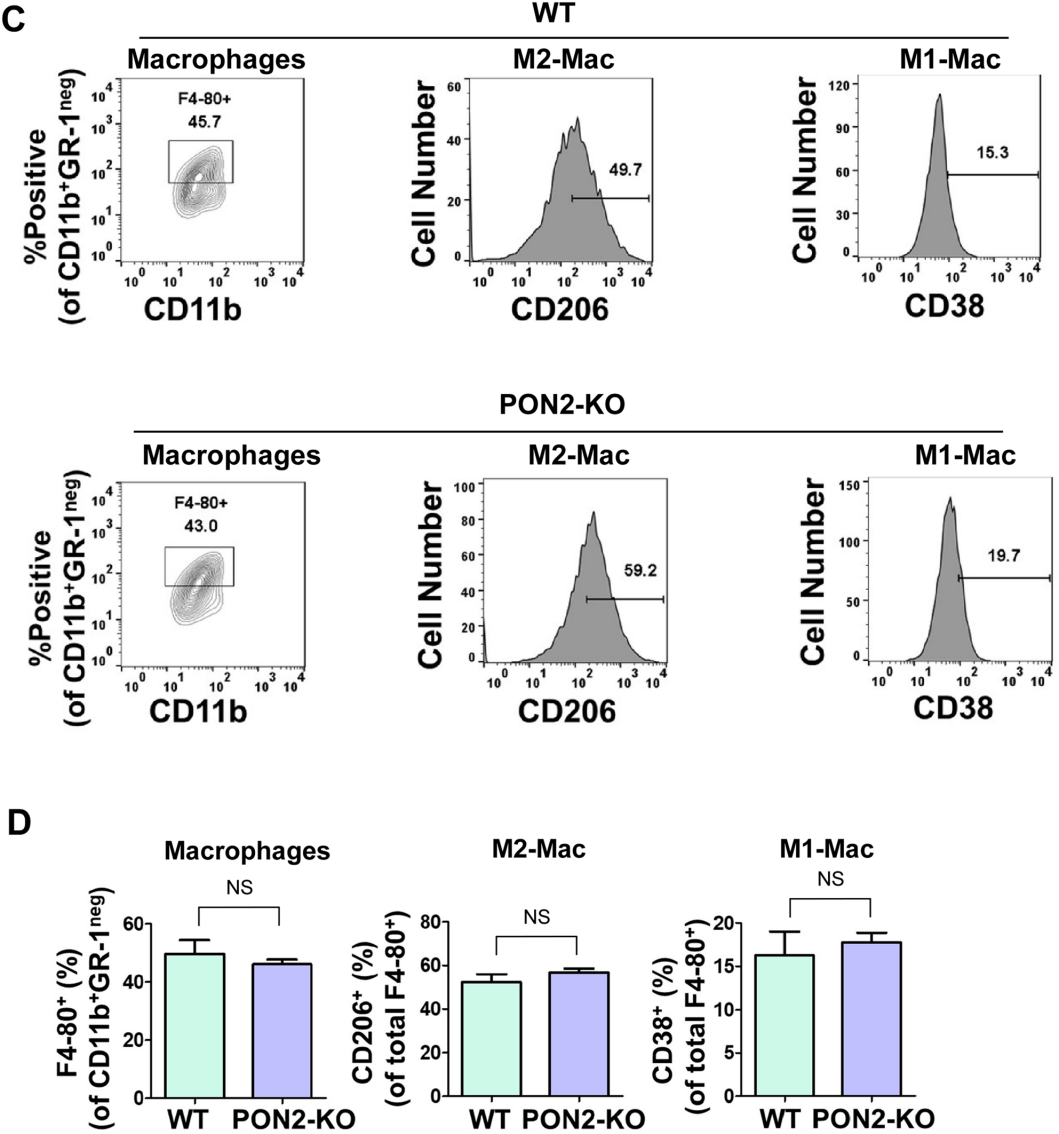
Host PON2 status does not influence the frequencies of MDSCs and the polarization states of macrophages in LLC tumors. Female WT or PON2-KO mice (n = 3 mice/group) were subcutaneously injected with 2 × 10^5^ LLC cells and euthanized 21 days after tumor challenge. Subsequently, tumors were removed and enzymatically digested to generate a single cell suspension. Tumor-infiltrating cells from WT and PON2-KO mice were stained with antibodies against surface markers to identify G-MDSCs, M-MDSCs, M1- and M2-macrophages, and analyzed by flow cytometry. Tumor-infiltrating immune cells were identified by CD45, a pan-hematopoietic marker. (**A**) Representative dot plot evaluation of intratumoral G-MDSCs (CD45^+^CD11b^+^Gr-1^hi^) and M-MDSCs (CD45^+^CD11b^+^Gr-1^int^). (**B**) Summary of the data presented in (**A**). Bar graphs show the percentages of CD45^+^ cells in total live cells, CD11b^+^ cells in CD45^+^ cells, Gr-1^hi^ G-MDSCs in CD45^+^CD11b^+^ cells, and Gr-1^int^ M-MDSCs in CD45^+^CD11b^+^ cells, respectively. Data are mean ± SEM of n = 3 mice in WT or PON2-KO group. NS = no significance. (**C**) Representative contour plot analysis of macrophages (CD45^+^CD11b^+^Gr-1^neg^F4-80^+^) and histogram analysis of M1-macrophages (CD45^+^CD11b^+^Gr-1^neg^F4-80^+^CD38^+^) and M2-macrophages (CD45^+^CD11b^+^Gr-1^neg^F4-80^+^CD206^+^). (**D**) Bar graphs showing the percentages of intratumoral F4-80^+^ macrophages in CD45^+^CD11b^+^Gr-1^neg^ cells, CD206^+^ cells in CD45^+^CD11b^+^Gr-1^neg^F4-80^+^ macrophage population (M2-Mac), and CD38^+^ cells in CD45^+^CD11b^+^Gr-1^neg^F4-80^+^ macrophage population (M1-Mac), respectively. Data are presented as mean ± SEM with 3 mice in WT or PON2-KO group. *NS* no significance.

It has been shown that tumor-infiltrating lymphocytes (TILs) play critical roles in cancer initiation and progression^33^. To explore the impacts of PON2 expression in mice on tumor-infiltrating myeloid cells, we examined the phenotypic characteristics of two major types of myeloid cells present within the tumors: myeloid-derived suppressor cells (MDSCs)^34^ and tumor-associated macrophages^35^, in subcutaneous LLC-bearing WT and PON2-KO mice (Fig. 8). The relative abundances of the two subsets of MDSCs, monocytic myeloid-derived suppressor cells (M-MDSCs) and granulocytic myeloid-derived suppressor cells (G-MDSCs), were comparable regardless of host’s PON2 expression status (Fig. 8A,B). Furthermore, the frequencies of macrophages were similar in LLC tumors from WT or PON2-KO mice (Fig. 8C,D). Finally, no significant differences were detected between WT and PON2-KO mice with respect to the abundance of tumor-infiltrating M1 and M2 subtypes of macrophages (Fig. 8C,D). Together, these observations indicate that host PON2 expression does not influence MDSC abundance and macrophage phenotypes present within subcutaneously transplanted LLC tumors.

## Discussion

In the present study, we investigated PON2’s role in lung cancer cell proliferation and lung tumorigenesis. PON2 was found to be essential for the proliferation of both murine and human lung tumor cells in vitro (Figs. 1 and 2), probably through promoting mitochondrial oxidative metabolism (Table 1). In contrast to normal eukaryotic cells, which meet their energetic demands through mitochondrial oxidative phosphorylation, most cancer cells instead preferentially undergo glycolysis to fuel their rapid proliferation, even under aerobic conditions. This phenomenon, “the Warburg effect,” was described nearly 100 years ago by Otto Warburg and was hypothesized to be a consequence of impaired mitochondrial function^36^. Subsequent studies in the past decades have illustrated that cancer cells have intact mitochondrial function and, instead, reprogram their metabolism to elevate so-called “aerobic glycolysis” as an adaptive mechanism to generate nucleotides, amino acids, and fatty acids sufficient for cell division^36^. This means that mitochondrial function is not expendable for cancer cell proliferation. For example, oncogenic transformation of human mesenchymal stem cells increases their dependency on mitochondrial oxidative metabolism for energy production^37^, and mitochondrial oxidative metabolism is critical for oncogenic Kras-driven cell proliferation and mouse lung adenocarcinoma tumorigenesis^38^. Herein, we have systematically evaluated major aspects of cellular bioenergetics in cultured lung adenocarcinoma cells with or without endogenous PON2 expression (Supplementary Figs. 2–4; Table 1). These studies have provided comprehensive information about metabolic changes caused by deficiency in PON2 expression, thus validating the pivotal role of PON2 in maintaining oxidative metabolism in cultured human lung adenocarcinoma cells. As such, one of major conclusions of this study is that elevated PON2 expression serves to promote cellular proliferation in vitro by enhancing lung adenocarcinoma mitochondrial bioenergetics.

Here we also explored the involvement of PON2 in pulmonary oncogenesis by examining PON2-proficient or PON2-deficient immunocompetent mice of both sexes. We sought to elucidate how the interplay between PON2 expression in transplanted lung tumors and host mice influences the neoplastic progression of lung tumors. In the three lung tumor models examined, PON2 expression in subcutaneously or orthotopically transplanted tumors and primary lung adenocarcinoma driven by oncogenic Kras as well as in host mice failed to significantly impact the initiation and development of tumors. Clearly, there is a discrepancy between PON2’s roles in cancer cell proliferation in vitro and lung tumor progression in vivo. Although cancer cell culture models provide valuable tools for understanding cancer progression and treatment, the physiological relevance of in vitro cell cultures is limited^39^. Therefore, the results of in vitro tissue culture experiments are unable to be directly extrapolated to in vivo animal studies, particularly for the complex and dynamic pathophysiological processes underlying tumorigenesis. It is not uncommon that the findings from cultured cancer cells are inconsistent with corresponding studies in animals^40^.

An important aspect of this study was to examine the functional characteristics of macrophages and MDSCs present in the tumor microenvironment (TME) of mice with or without PON2 expression. Our research revealed that in mice implanted with subcutaneous LLC tumors, neither the polarization state of macrophages nor relative abundance of MDSC subtypes present in the TME was influenced by host PON2 expression (Fig. 8), which is consistent with our finding that PON2 does not play a role in implanted LLC tumor development (Fig. 4). Macrophages have a high degree of phenotypic plasticity along a continuum from the pro-inflammatory M1 macrophages that defend against foreign pathogens and neoplastic cells, to the anti-inflammatory M2 macrophages that support tissue homeostasis and repair^34^. M2 macrophages are the most abundant immune cell subtypes present in the lung tumors and have been shown to promote tumorigenesis^41^. An earlier study demonstrated that PON2 expression enhanced M2 polarization phenotypes in glycollate-elicited murine peripheral macrophages (MPMs) through examining ex vivo MPM responses following exposures to single cytokines^22^. In the in vivo TME niche, a multitude of competing pro- and anti-inflammatory signals are present that crosstalk with tumor cells, which may explain the discordance between the present study and the previous report^22^.

The noncancerous cellular and noncellular factors surrounding tumors are collectively known as TME^42,43^. The vital components of the TME include fibroblasts, neuroendocrine cells, adipocytes, immune cells, vasculature, lymphatic networks, and extracellular matrix (ECM). Accumulating evidence indicates that TME plays an essential role in the initiation, progression, and metastasis of human tumors^44,45^. Tumor physiology is modulated by different aspects of the tumoral niche, including intrinsic properties of tumor cell metabolism, genetic heterogeneity, metabolic interplays between tumor cells and nontumorous cells, oxygen availability, as well as whole-body nutrient homeostasis^46,47^. In contrast, in vitro cell culture models rely on studying single homogenous cell types cultured in the presence of abundant oxygen and nutrients uncomplicated by the involvement of TME components. Thus, significant differences have been discovered between the metabolism of tumors and the metabolism of corresponding malignant cells cultured in vitro^48^. These considerations may account for the discrepancy between PON2’s role in the proliferation of cultured lung cancer cells and the progression of murine lung tumors.

Given the heterogenicity of intrinsic (e.g., oncogenic profiles of neoplastic cells) and extrinsic properties (e.g., nutrient supply and oxygen availability) of the tumor niche, the effects of PON2 on tumorigenesis are likely vastly different among various cancer types. Recent research efforts have revealed diverse underlying mechanisms of PON2 expression impacting tumorigenesis. In PDAC cells, PON2 promoted cellular proliferation by enhancing GLUT1-mediated glucose transport, thereby protecting against the cellular starvation response and promoting anoikis^14^. An important finding of this work is that PON2 is transcriptionally repressed by the tumor suppressor p53, providing mechanistic insight into how tumors may favorably upregulate PON2 expression. Likewise, studies of PON2-deficient murine and human B-ALL cells revealed that the loss of PON2 resulted in compromised leukemogenesis through defective glucose uptake and ATP production^10^. In accordance with the study conducted in PDAC cells^14^, PON2 in B-ALL cells enabled glucose uptake by preventing stomatin-mediated inhibition of GLUT1. On the other hand, overexpression of PON2 in an ovarian cancer cell line decreased cellular proliferation via the inhibition of IGF-1 expression and signaling^15^. This phenotype was due to PON2’s ability to prevent mitochondrial superoxide generation, which increases c-Jun-mediated transcriptional activation of the IGF-1 gene. Taken together, these results paint a complicated picture of PON2’s role in tumorigenesis broadly and implicate its diverse roles in different tumor types.

It is conceivable that the interaction of lung tumors and microenvironmental factors in our animal studies are distinct from those of PDAC, B-ALL, and ovarian cancer, yielding different impacts of PON2 on tumorigenesis. Overall, prudence is required for extrapolating in vitro cancer cell studies to animal research. As such, future exploration is warranted to further elucidate the role of PON2 in initiation, progression, and metastasis of lung cancer.

## Materials and methods

### Reagents and antibodies

Dulbecco’s Modified Eagle’s Medium (DMEM), penicillin/streptomycin, trypsin, and L-glutamine were obtained from Mediatech (Manassas, VA). Fetal bovine serum (FBS) was purchased from Gemini (Broderick, CA). Polybrene, *N*-(3-oxododecanoyl)-homoserine lactone (C12), puromycin, [U-^13^C]-glucose, deuterium oxide (D_2_O), 2-Dimethyl-2-silapentane-5-sulfonate sodium salt (DSS), collagenase, and heparin were purchased from Sigma-Aldrich (St. Louis, MO). Lipofectamine2000^®^ transfection reagents, propidium iodide, β-Mercaptoethanol and Matrigel^®^ were purchased from Thermo Fisher (Waltham, MA). Antibodies (Abs) for western blot were anti-β-actin mAb (A5441; Millipore Sigma; Burlington, MA), anti-Mouse PON2 pAb (ABIN1573944; antibodies-online.com; Atlanta, GA), peroxidase-conjugated goat anti-rabbit IgG (65-6120; Thermo Fisher; Waltham, MA) and peroxidase-conjugated goat anti-mouse IgG (65-6520; Thermo Fisher). Ad5-CMV-Cre was purchased from the Baylor College of Medicine Gene Vector Core.

### Cell culture

Human Bronchial Epithelial (HBE) cells immortalized by hT/LT were obtained from Professor Barrett Rollins at Harvard Medical School^49^. HBE cells were grown in Bronchial Epithelial Cell Growth Medium (BEGM™) supplemented with SingleQuots (LONZA; Morristown, NJ). Lewis Lung Carcinoma, A549, NCI-H1299, and HEK-293T cells were purchased from ATCC (Manassas, VA), and they were cultured in DMEM containing 10% FBS, 100 units/mL penicillin, and 100 μg/mL streptomycin. All the cells are cultured in a 5% CO_2_ humidified incubator at 37 °C. Cells were passaged at approximately 1:5–1:10 dilutions and were continuously cultured no longer than 3 weeks. Stock from thawed vials were frozen at passage two following the receipts from supplies. LLC, A549, NCI-H1299, and 293T cells were authenticated by ATCC cell bank using the Short Tandem Repeat (STR) profiling.

### Plasmids

Control double nickase plasmid (sc-437281) and PON2 Double Nickase Plasmid (sc-403181-NIC) were purchased from Santa Cruz (Dallas, TX). Control shRNA plasmid-A (SC108060), murine PON2-shRNA plasmids (sc-62839-SH) and human PON2-shRNA plasmids (SC62838-SH) were purchased from Santa Cruz. Lentiviral helper plasmids pMDLg/pRRE (12251), pRSV.Rev (12253), and pMD2.G (12259) were purchased from Addgene (Watertown, MA). The retroviral plasmid pBABE-IRES-EGFP (14,430) as well as retroviral helper plasmids pUMVC (8449) and pMDLg/pRRE (12251) were also acquired from Addgene. The identities of each plasmid were verified by sequencing.

### Generation of cells with reduced PON2 expression

To produce lentivirus, HEK-293 T cells (1.5 × 10^6^) were plated in 6-cm tissue culture plates, cultured for 24 h, then transfected with control shRNA plasmid-A (SC108060), murine PON2-shRNA plasmids (sc-62839-SH), or human PON2-shRNA plasmids (SC62838-SH) along with the lentiviral helper plasmids pMDLg/pRRE, pRSV.Rev, and pMD2.G using Lipofectamine2000® transfection reagent (Thermo Fisher). Tissue culture medium containing lentiviral particles was collected 48 and 72 h following transfection and was filtered through a sterile syringe filter with 0.4 μM polyethersulfone membrane (VWR; Radnor, PA). LLC, A549, HEK-293 T, and HBE cells were seeded 24 h prior to lentiviral infection in 6-well tissue culture plates. For lentiviral infection, culture medium of plated cells was replaced with medium containing lentiviral particles supplemented with 10 μg/mL polybrene; infection was repeated 24 h later. The day following the second infection, puromycin (final concentration 5 μg/mL) was added to culture medium to eradicate uninfected cells. For all subsequent experiments, cells were cultured in medium containing 1 μg/mL puromycin. PON2 protein expression in infected cells was examined by western blot.

For retrovirus production, HEK-293T cells were transfected with the retroviral plasmid pBABE-IRES-EGFP along with the helper plasmids pUVMC and pMD2.G using the transfection reagent Lipofectamine2000^®^ (Thermo Fisher). Retroviral supernatant was collected 48 and 72 h after transfection with the addition of 10 μg/mL polybrene to increase infection efficiency. GFP protein was expressed in LLC cells expressing control- or PON2-shRNA by culturing cells with retrovirus-containing medium. Nearly all infected cells (> 95%) were GFP positive as evaluated by flow cytometry (FACScalibur; Beckon Dickinson; San Jose, CA).

### Elimination of endogenous PON2 expression by CRISPR/Cas9

NCI-H1299 cells (2 × 10^6^) were plated in 10-cm tissue culture plates and cultured for 24 h. Control double nickase plasmid (SC-437281) and PON2 CRISPR/Cas9 plasmid (SC-403181-NIC) were transfected into NCI-H1299 cells using Lipofectamine2000® transfection reagent (Thermo Fisher) according to manufacturer’s protocol. Puromycin (5 μg/mL) was added to culture medium to select against untransfected cells 24 h following transfection. Cells were maintained in culture medium supplemented with 5 μg/mL puromycin for 3 weeks to allow stably transfected cells to proliferate. Fluorescence-activated cell sorting (FACS; MoFlo, Beckman Coulter, Brea, CA) was used to establish single clones of stably transfected cells by seeding GFP-positive cells into individual wells of 96-well tissue culture plates containing medium supplemented with 5 μg/mL puromycin. Western blot analysis was then used to detect PON2 expression in clonal cell lines. For subsequent experiments, clonal cells were cultured in medium containing 1 μg/mL puromycin.

### Western blot analysis

Whole cell extracts were prepared using RIPA buffer (Thermo Fisher) supplemented with protease inhibitors (cOmplete; Roche; Indianapolis, IN). Protein concentration was measured by bicinchoninic acid (BCA assay; Thermo Fisher). Total protein (30 μg) was electrophoresed in 4–20% Bis/Tris gels (Genscript; Piscataway, NJ). Separated proteins were transferred to a polyvinylidene difluoride (PVDF) membrane (Millipore) and incubated with indicated primary or secondary antibodies in blotting buffer containing 1X phosphate-buffered saline (PBS), 0.2% Tween-20, and 10% (w/v) non-fat dry milk (Thermo Fisher). The enhanced chemiluminescence detection system (Thermo Fisher) was used to detect proteins as previously described^19^.

### Cell death assays

The indicated cells were plated in 48-well tissue culture plates with 10,000 cells in each well and cultured for 24 h. Unless otherwise stated, medium containing 0.1% DMSO with or without the indicated agents was incubated with the cells. Following treatment with the indicated agents, cells were treated with trypsin and collected with addition of final concentration of 1 µg/mL PI. Cell viability was determined via PI exclusion using flow cytometry as described earlier^19^. The percentage of cell death was calculated as100 minus the value of cell viability measurement. The values of triplicate samples from three individual wells of a 48-well tissue culture plate were acquired and averaged. Data are presented as mean ± SD of 3 independent experiments.

### Caspase-3/7 activity

Caspase-3/7 activity was determined using a Caspase-Glo^®^ 3/7 Assay kit (Promega; Madison, WI) following manufacturer’s protocol. Twenty-four hours before the treatment, LLC cells were plated in white-walled 96-well plates (5 × 10^3^ cells/well). Cells were treated with either 0.1% vehicle control (DMSO) or the indicated concentrations of C12 and incubated at 37 °C in a 5% CO2 humidified incubator. Two hours following treatment with agents, an equal volume of Caspase-Glo^®^ reagent was added to each well and equilibrated for 1 h, and luminescence was measured using a SpectaMax iD3 microplate spectrofluorometer (Molecular Devices; Sunnyvale, CA) according to manufacturer’s protocol. Data were presented as relative luminescence units (RLUs). The values of duplicate samples from two individual wells of a 96-well plate were acquired and averaged. Data are shown as mean ± SD of fold-change in relative luminescence units (RLU) based on 3 independent experiments.

### Cellular proliferation experiments

For cell proliferation measurement, LLC cells (0.5 × 10^4^/well), A549 cells (1.5 × 10^4^/well), NCI-H1299 cells (1.5 × 10^4^/well), HEK-293 T cells (1.5 × 10^4^/well), and HBE cells (1.5 × 10^4^/well) were plated in 12-well tissue culture plates and the number of the cells in each well was determined using a hemocytometer 24, 48, 72, and 96 h after plating. The values of triplicate samples from three individual wells of a 12-well plate were acquired and averaged. Data are presented as mean ± SD of 3 independent experiments.

### Cell cycle analysis

LLC, A549 and NCI-H1299 cells (5 × 10^5^) were collected and centrifuged at 300 × g for 5 min and washed twice with 500 µL 1X PBS. Cells were then fixed overnight in 1 mL 70% ethanol in 1X PBS. After centrifugation (300×*g* for 5 min), cells were washed twice with 1X PBS and resuspended in 500 µL 1X PBS. Cells were incubated with 50 U RNase A (Qiagen; Valencia, CA) at 37 °C for 1 h. Propidium iodide (5 μg) was added to each sample and incubated for an additional 30 min at 37 °C, after which PI stain intensity was measured using flow cytometry (FACScalibur). Data were analyzed using FloJo software (Beckon Dickinson).

### NMR sample preparation

NCI-H1299 vector-CRISPR or PON2-CRISPR (6 × 10^5^) cells were plated in 15-cm cell culture dishes with DMEM medium containing 10% glucose-free FBS and 5 mM [U-^13^C]-glucose. Glucose-free FBS was acquired by dialyzing regular FBS against 1X PBS three times using dialysis membrane with molecular weight cutoff of 12–14 kD (Thermo Fisher). At 0-, 24-, 48- and 72-h timepoints, the culture medium was collected, flash frozen in liquid nitrogen, and stored at − 80 °C. At 72-h timepoint, Cells were treated with trypsin, collected by centrifugation, and washed 3 times with 20 mL ice-cold 1X PBS. Cell pellets (7 × 10^6^) were flash frozen in liquid nitrogen and stored at − 80 °C. To extract metabolites, 200 μL growth medium sample was mixed with 200 μL 40% ice-cold trichloroacetic acid (TCA) and 300 μL 10% TCA was added to cell pellets. Samples were centrifuged to remove precipitates and supernatants were lyophilized as previously described^21,50^.

### NMR analysis

The lyophilized extracts for medium and cells were dissolved in 100% D_2_O (0.35 mL) and loaded into a 5 mm Shigemi NMR tube (Sigma-Aldrich). DSS was added to the samples at a final concentration of 50 nM to serve as a ^1^H reference standard. NMR spectra were recorded at 14.1 T on a Varian Inova spectrometer (Palo Alto, CA) equipped with a 5 mm inverse triple resonance cold probe at 20 °C. 1D NMR spectra were recorded with an acquisition time of 2 s and a recycle time of 5 s. Concentrations of metabolites and ^13^C incorporation were determined by peak integration of the ^1^H NMR spectra referenced to the DSS methyl groups, with correction for differential relaxation, as previously described^21,50^. ^1^H Spectra were typically processed with zero filling to 131 k points, and apodized with an unshifted Gaussian and a 0.5 Hz line broadening exponential. ^13^C profiling was achieved using 1D ^13^C-edited HSQC ^1^H spectra recorded with a recycle time of 1.5 s, with ^13^C GARP decoupling centered at 80 ppm (1JCH set to 150 Hz) during the proton acquisition time of 0.15 s.

TOCSY spectra were recorded with a mixing time of 50 ms and a B1 field strength of 8 kHz with acquisition times of 0.573 s in t_2_ and 0.036 s in t_1_. The fids were zero filled once in t_2_, and linear predicted and zero filled to 4096 points in t_2_. The data were apodized using a squared cosine bell function in both dimensions. Specific ^13^C isotopomers and fractional incorporation of ^13^C were determined by comparing the areas or volume of satellite peaks with the total integrated area or volume, with appropriate corrections for differential relaxation as previously described^21,50^.

### Ethics statement

Mice were handled in accordance with the American Association for the Accreditation of Laboratory Animal Care (AAALC) guidelines and the “Guide for the Care and Use of Laboratory Animals” (Institute of Laboratory Animal Resources, National Research Council, National Academy Press, 1996). The mouse study was approved by the Institutional Animal Care and Use Committee (IACUC) of the University of Louisville (protocol numbers: 18192 and 20860).

### Mouse strains

Mice were housed in an AALAC-accredited pathogen-free barrier facility. All procedures were conducted in accordance with the University of Louisville IACUC guidelines. Eight-week-old male and female Kras^LSL-G12D^ (B6.129S4-Kras^tm4Tyj^/J; 008179) mice were purchased from Jackson laboratories (Bar Harbor, ME). PON2-KO mice were described in our earlier studies^19^. Kras^LSL-G12D^ mice were selectively bred with PON2-KO mice to generate PON2-heterozygous mice harboring the mutant Kras^LSL-G12D^ allele. These mice were subsequently bred to generated mice homozygous for either wild-type PON2 or mutant PON2 alleles that also possessed a single Kras^LSL-G12D^ allele. PON2 genotypes were validated by Sanger sequencing of mouse genomic DNA as described earlier^19^. Kras^LSL-G12D^ genotypes were confirmed by PCR amplification of genomic DNA using the following primers: Kras y116-common, 5′-TCC GAA TTC AGT GAC TAC AGA TG-3′; Kras y117-LSL, 5′-CTA GCC ACC ATG GCT TGA GT-3′; and Kras y118-wt, 5′-ATG TCT TTC CCC AGC ACA GT-3′. PCR reaction conditions are as follows: 35 cycles of 95 °C for 30 s, 60° C for 30 s, and 72 °C for 30 s. PCR products were resolved on a 2% agarose gel. The expected product size of y117/y116 is 327 bp (LSL), and y116/y118 is 450 bp (wild-type).

### Subcutaneous tumor injection

LLC cells (control- and PON2-shRNA; 1 × 10^5^ cells in 100 μL sterile 1X PBS) were injected subcutaneously into the right flank of wild-type and PON2-KO mice. Tumor volume was measured every other day using dull-edged Vernier calipers (V = L*W^2^/2, where V is volume, L is length, and W is width). Per IACUC guidelines, experimental endpoint was reached when tumors exceeded 1000 mm^3^ in volume. At endpoint, mice were euthanized via CO_2_ inhalation.

### Orthotopic allograft lung tumor model

GFP-positive LLC cells expressing either control- or PON2-shRNA were percutaneously injected into the pleural cavity of wild-type or PON2-KO mice of both sexes according to a published report, with minor modifications^26^. Briefly, mice were anesthetized via inhalation of vaporized isoflurane (3% with 1.5 L/min oxygen flow). Anesthetic depth was confirmed by toe pinch reflex. Cells (2 × 10^5^) were suspended in 1:1 PBS:Matrigel and percutaneously injected into the left lateral thorax using 1-mL syringes with 26-gauge hypodermic needles. After 10 days, mice were euthanized by CO_2_ inhalation. Thoracic cavity was exposed using surgical instruments, and lungs were perfused by injecting 10 mL 1X PBS supplemented with 10U/mL heparin into the right ventricle of the heart using a 22-guage needle. Perfused lung and tumor tissues were dissected and minced with surgical scissors. Minced tissues were incubated for 30 min in 10 mL 0.25% trypsin–EDTA supplemented with 300 U/mL collagenase in a 37 °C water bath. Suspension was agitated using a 1000 μL pipette, followed by a 22-gauge needle, and passed through a 0.4 μm filter. GFP positivity of single cell suspension was determined by flow cytometry (FACScalibur).

### Mutant Kras-driven primary lung tumor model

Details of the Kras^LSL-G12D^ mouse model of primary lung tumorigenesis have been previously described^51^. Tumor initiation was accomplished by intratracheal instillation of adenovirus expressing Cre-recombinase (2.5 × 10^7^ PFU/mouse). Briefly, mouse anesthesia was initiated and maintained via the inhalation of vaporized isoflurane (3% in 1.5 L/min O_2_). Anesthetic depth was confirmed by the absence of toe reflex prior to surgery. Once anesthetized, mice were subcutaneously administered 200 μL meloxicam (0.5 mg/mL). Surgery was performed on a slide warmer covered with sterile paper towels and maintained at 37 °C. Mice were affixed with surgical tape to expose the ventral surface, and Nair depilatory cream was applied to remove hair at surgical site. Exposed area was swabbed with alternating applications of iodine followed by isopropyl alcohol for three cycles. Tracheas were exposed using sterile surgical instruments, and Ad-Cre (2.5 × 10^7^ PFU/mL in 60 μL DMEM supplemented with 10 mM CaCl_2_) was administered with an insulin syringe directly into the tracheal lumen. Surgical incision was closed with surgical staples, and anesthesia was withdrawn to allow mice to recover. Mice were subcutaneously administered meloxicam 200 μL meloxicam (0.5 mg/mL) 24 and 48 h after surgery. Mice were euthanized 28 weeks after tumor initiation, and lung tissues were collected for histological examination.

### Histological analysis of lung tissues

Lung tissue sections were prepared as previously described, with some modifications^52^. Briefly, lung tissues were fixed in 10% neutral phosphate buffered formalin for 24 h at room temperature. After paraffin processing (TEK VIP; Sakura Finetek; Torrance, CA) and embedding (EG1160; Leica Biosystems; Buffalo Grove, IL), paraffin microtomy (RM2135; Leica Biosystems) was performed at 5 μM thickness per section. 5 sections were processed with 200 μM between each to assure the detection of tumorigenesis in a whole lung. Slides of sections were deparaffinized and rehydrated in xylene, ethanol, and deionized water. Slides were stained with hematoxalin (95057-844; VWR) and eosin (HT110232; Thermo Fisher) according to a published protocol^52^. Stained slides were dehydrated in ethanol and xylene and coverslips were affixed to slide using xylene-based Permount™ mounting medium (SP15-500; Thermo Fisher; Waltham, MA). Slides were dried overnight in a chemical hood, scanned using an Aperio Imagescope (Leica Biosystems). CaseViewer software (3DHISTECH; Ramsey, NJ) was used to outline and measure lung/tumor cross-sectional area. Tumor burden was calculated for all sections as the sum of tumor cross-sectional area divided by the cross-sectional area of the whole lung section.

### Flow cytometry analysis of immune cells

Female wild-type or PON2-KO mice (8-week-old) were subcutaneously implanted with 2 × 10^5^ LLC cells. Tumor-bearing mice were euthanized on day 21 following LLC implantation. Tumor tissues were harvested from mice, minced into small pieces with surgical scissors, and enzymatically digested with a mixture containing 400 U/mL collagenase type IV (C9891), 0.025 mg/mL hyaluronidase (H6254), and 0.01 mg/mL DNase I (D5025) purchased from Sigma-Aldrich for 2 h at 37 °C with occasional shaking. Acquired single cell suspensions were used for flow cytometry analysis. For surface staining, single cell suspensions were washed and resuspended in PBS, then stained with Fixable Viability Dye eFluor™ 450 (Thermo Fisher Scientific, USA) for 15 min. Cells were then washed twice and resuspended in FACS buffer [2% FBS + PBS] and stained with multi-color antibody (Ab) cocktails according to the manufacturer’s recommendations. Antibodies used in this study are provided in Supplemental Table 1. Murine cells were incubated with CD16/CD32 antibody (2.4G2) for 10 min at 4 °C to block Fc receptors before being stained for surface markers, washed, and resuspended in flow buffer. Stained cells were analyzed with a FACS BD LSRFortessa (Becton Dickinson [BD] Biosciences, Franklin Lakes, NJ) and data were analyzed using FlowJo v10 (BD Biosciences). FACS dot plots are represented with log-scale axes. Histograms are represented on a log-scale x-axis and a linear y-axis.

### Statistical analysis

Statistical significance was determined by Student’s t-test and/or two-way ANOVA as indicated. A *p*-value of < 0.05 was considered significant.

### ARRIVE reporting

The study is reported in accordance with ARRIVE guidelines.

## Supplementary Information

Supplementary Information.

## Data Availability

The authors confirm that the data supporting the findings of this study are available within the article and its supplementary materials.

## Abbreviations

PON2: Paraoxonase 2
PON2-KO: PON2-knockout
HBE: Human bronchial epithelial
HEK-293T: Human embryonic kidney-293T
BEGM: Bronchial epithelial cell growth medium
B-ALL: B cell acute lymphoblastic leukemia
PDAC: Pancreatic ductal adenocarcinoma
C12: *N*-(3-oxododecanoyl)-l-homoserine lactone
DMEM: Dulbecco’s modified Eagle’s medium
DMSO: Dimethyl sulfoxide
PI: Propidium iodide
SIRM: A stable isotope resolved metabolomics
NMR: Nuclear magnetic resonance
1D: One dimensional
2D: Two dimensional
TCA: The tricarboxylic acid
PPP: Pentose phosphate pathway
Ad-Cre: Adenovirus expressing Cre recombinase
TME: Tumor microenvironment
ECM: Extracellular matrix
FFPE: Formalin-fixed, paraffin-embedded
H&E: Hematoxalin and eosin
TILs: Tumor-infiltrating lymphocytes
M-MDSCs: Monocytic myeloid-derived suppressor cells
G-MDSCs: Granulocytic myeloid-derived suppressor cells
MPMs: Murine peripheral macrophages
