# Supplementary material for: Paraoxonase 2 (PON2) plays a limited role in murine lung tumorigenesis
Source: Sci Rep. 2023 Jun 19;13:9929. doi: 10.1038/s41598-023-37146-5 (PMC10279720; doi:10.1038/s41598-023-37146-5)

SUPPLEMENTARY FIGURE LEGENDS

Supplementary figure 1. The proliferation of nontransformed cells is not impacted by PON2 expression.

(A) PON2 expression was stably reduced in HEK-293T cells by RNAi and its levels were examined by western blot analysis. The molecular weight markers are labeled on the left (kD). **(B)** The viability of HEK-293T cells was measured 24 hours after C12 treatment. Data are mean ± SD of three independent experiments. **(C)** The proliferation of HEK-293T cells was measured daily by counting cells over the course of 4 days. Mean ± SD of three independent experiments is presented. **(D)** Stable decrease of PON2 expression in HBE cells was determined by western blot. The molecular weight markers are labeled on the left (kD). **(E)** The viability of HBE was evaluated 24 hours following C12 exposure. Mean ± standard deviation for three independent experiments is shown. **(F)** The proliferation of HBE cells was determined by cell counting. Data are Mean ± SD of three independent experiments. For all the data, asterisks indicate p-values of < 0.01 (**) by Student's unpaired t-test. NS = no significance.

Supplementary figure 2. Schematic depiction of NMR-based SIRM in NCI-H1299 cells.

The illustration of carbon flow from [U-^{13}C]-glucose into major intracellular metabolites through several metabolic processes, including glycolysis, the TCA cycle, pentose phosphate pathway, malate/aspartate shuttle, and biosynthesis of pyrimidine nucleotides. Expected ^{13}C isotopomers of metabolites are shown with open circles

representing ^{12}C and filled circles representing ^{13}C. Complex labeling patterns are a result of scrambling at the succinate step and subsequent turns of the TCA cycle.

Supplementary figure 3. Extracellular metabolites are evaluated by 1D high-resolution NMR.

Vector-CRISPR and PON2-CRISPR NCI-H1299 cells were cultured in medium containing [U-^{13}C]-glucose, which was collected at time 0 hour, and every 24 hours thereafter. The metabolites in the medium samples were detected by 1D ^{1}H NMR. The representative spectra of the medium collected at 0 **(A)** and 72-hour **(B & C)** timepoints are shown. The NMR signals of key metabolites, including $^{13}C\alpha$1-glucose, ^{12}C4H3-threonine, ^{12}C4H3-valine and ^{13}C3H3-lactate, are highlighted in blue.

Supplementary figure 4. Key ^{13}C-labeled soluble metabolites in NCI-H1299 cells are detected by SIRM.

Vector-CRISPR and PON2-CRISPR NCI-H1299 cells were cultured in medium containing 5 mM [U-^{13}C]-glucose for 72 hours. **(A)** ^{13}C-labeling of amino acids in NCI-H1299 cells was evaluated by TOCSY. The box “a” shows the ^{13}C satellite peaks of alanine, which surrounds the central peak representing unlabeled alanine. Similarly, Box “b” shows the satellite peaks of the C-2H and C-4H resonances of glutamate and the glutamate moiety of reduced glutathione, and box “c” shows the satellite peaks of aspartate C-2 and C-3. **(B)** Nucleotide labelling in vector-CRISPR and PON2-CRISPR NCI-H1299 cells was revealed by TOCSY. Box “a” shows the ^{13}C satellite peaks of the

ribose moieties of UTP. Box “b” shows doubly labelled uracil as well as the two singly labelled species derived directly from aspartate. All boxes are highlighted in red.

Supplemental Table 1. Mouse antibodies used in the study of tumor-infiltrating immune cells.

The details information about the antibodies used for the experiments of examining intratumoral MDSCs and macrophages.

Whitt et al., Supplementary figure 1

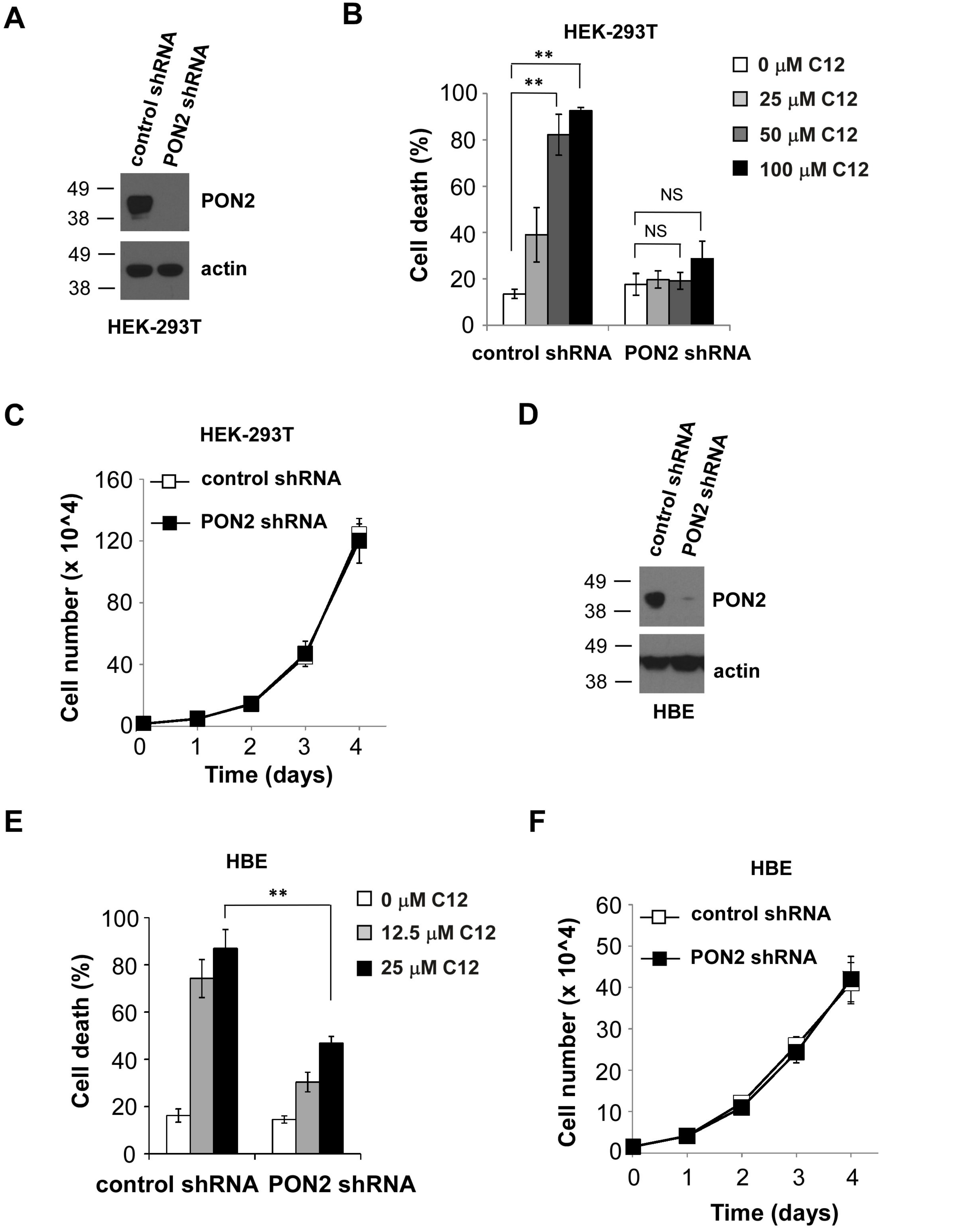

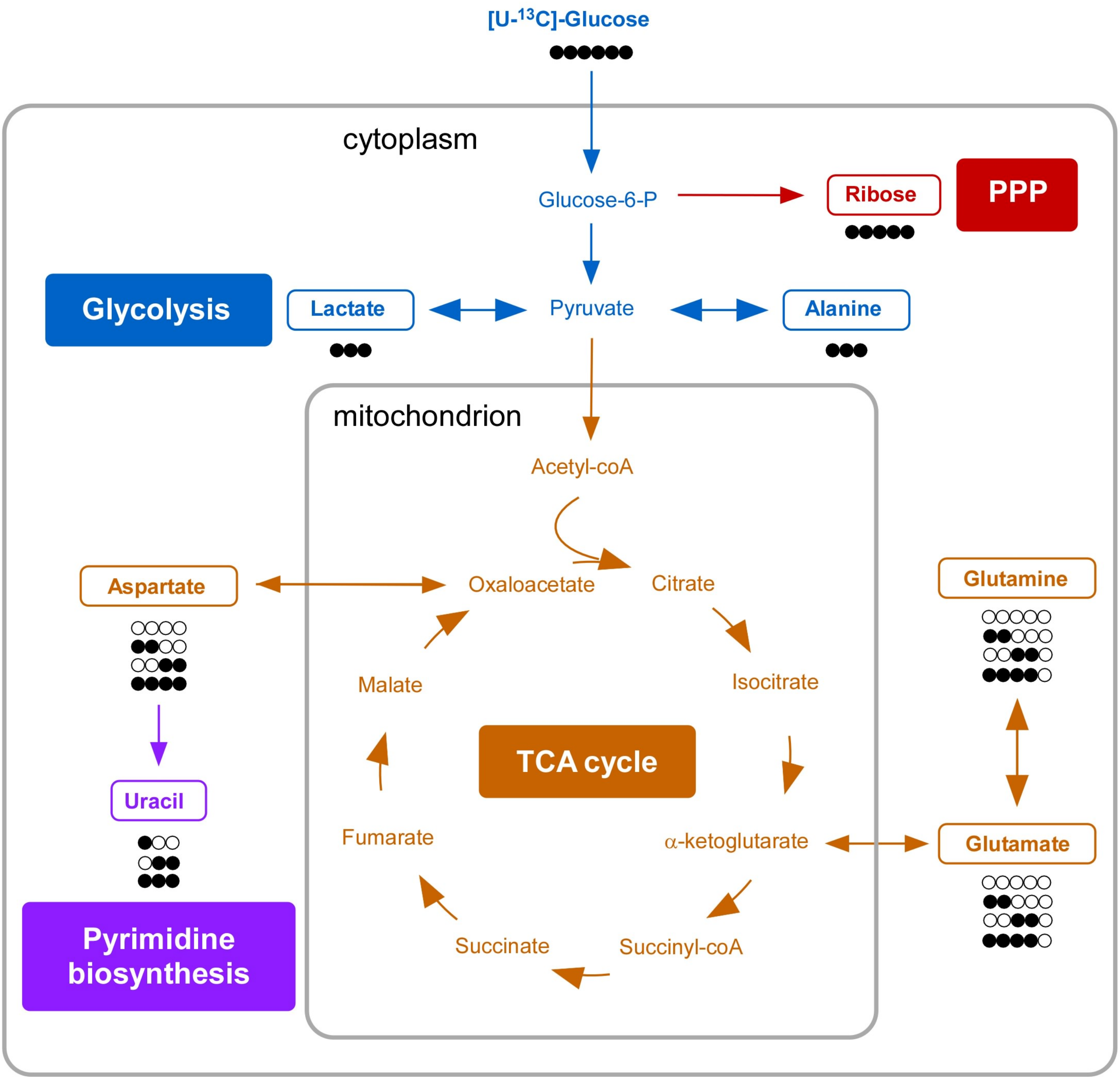
[U-13C]-Glucose
cytoplasm
Glucose-6-P
Ribose
PPP
Glycolysis
Lactate
Pyruvate
Alanine
mitochondrion
Acetyl-coA
Aspartate
Oxaloacetate
Citrate
Glutamine
Malate
Isocitrate
TCA cycle
Uracil
Fumarate
α-ketoglutarate
Glutamate
Succinate
Succinyl-coA
Pyrimidine biosynthesis

Whitt et al., Supplementary figure 3

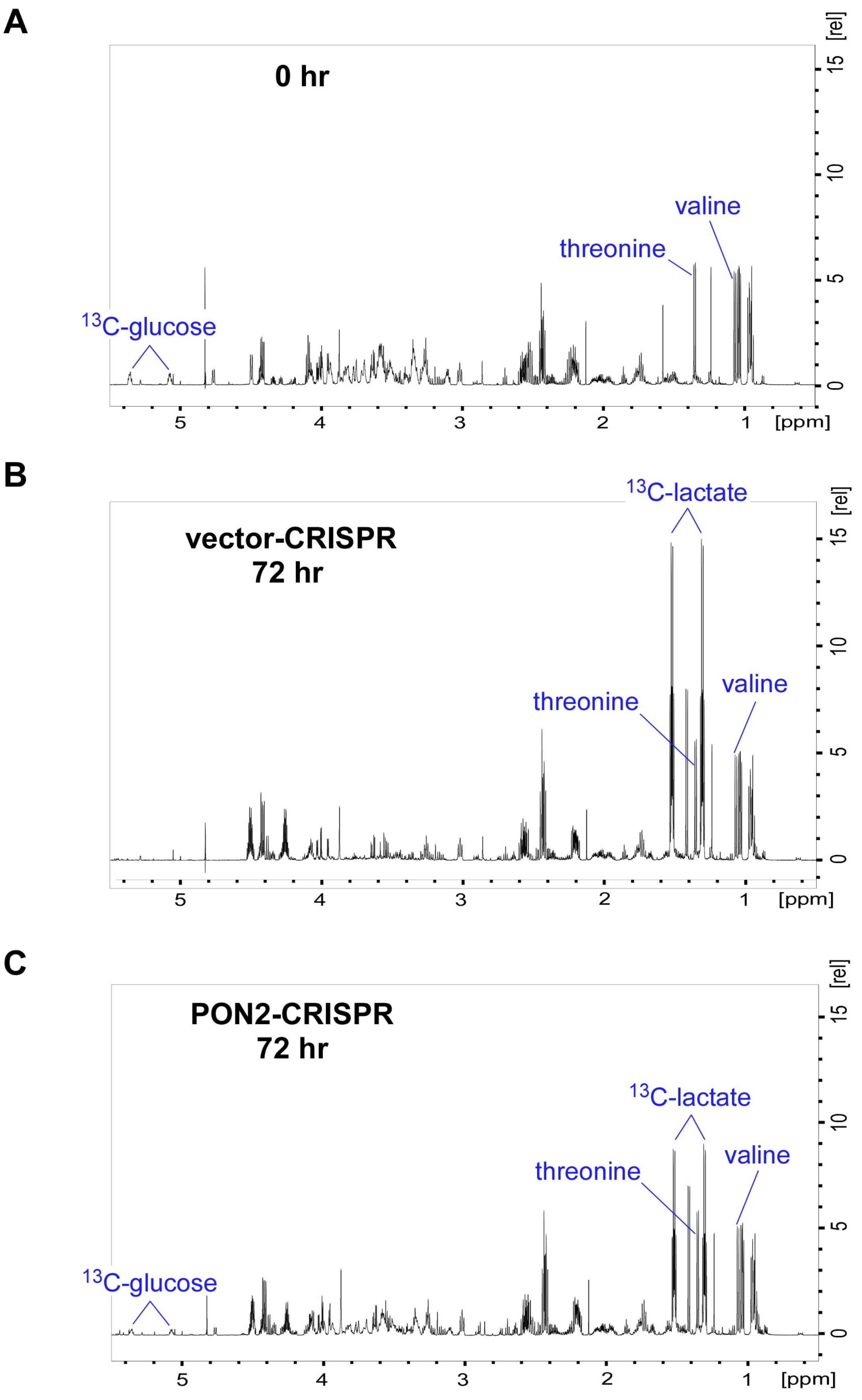

Whitt et al., Supplementary figure 4

A

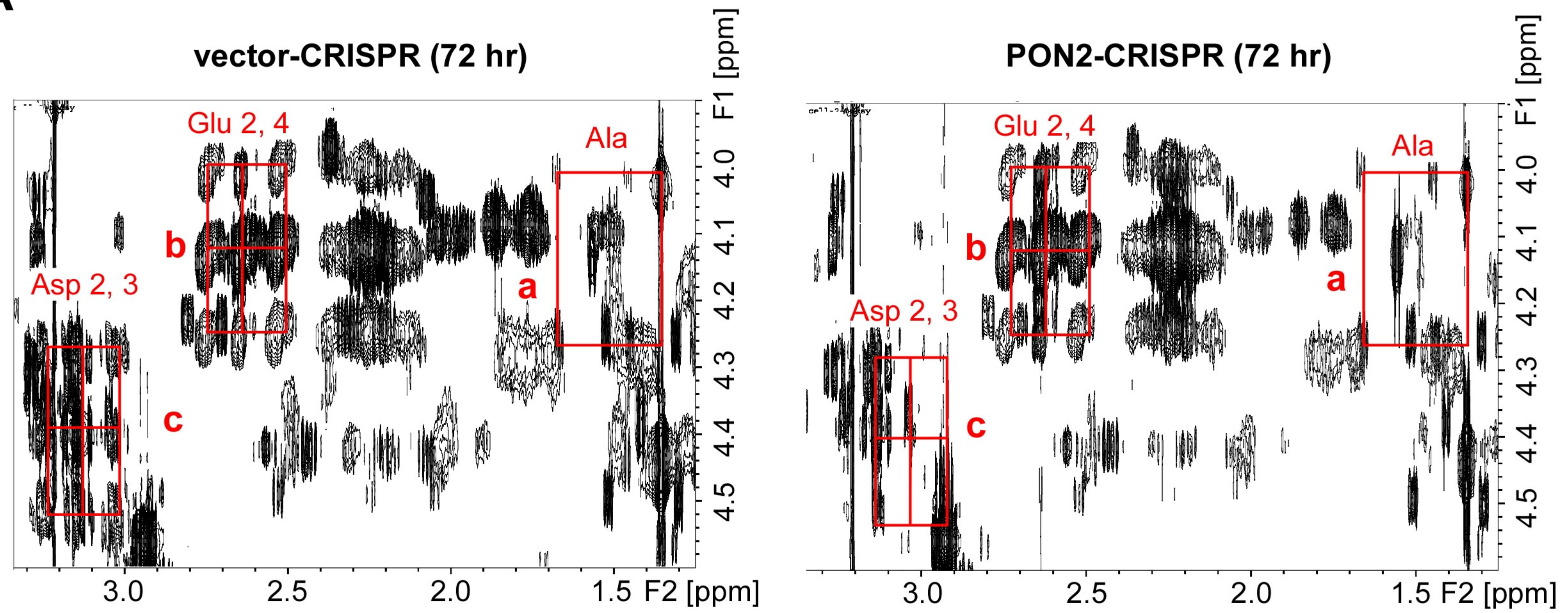

B

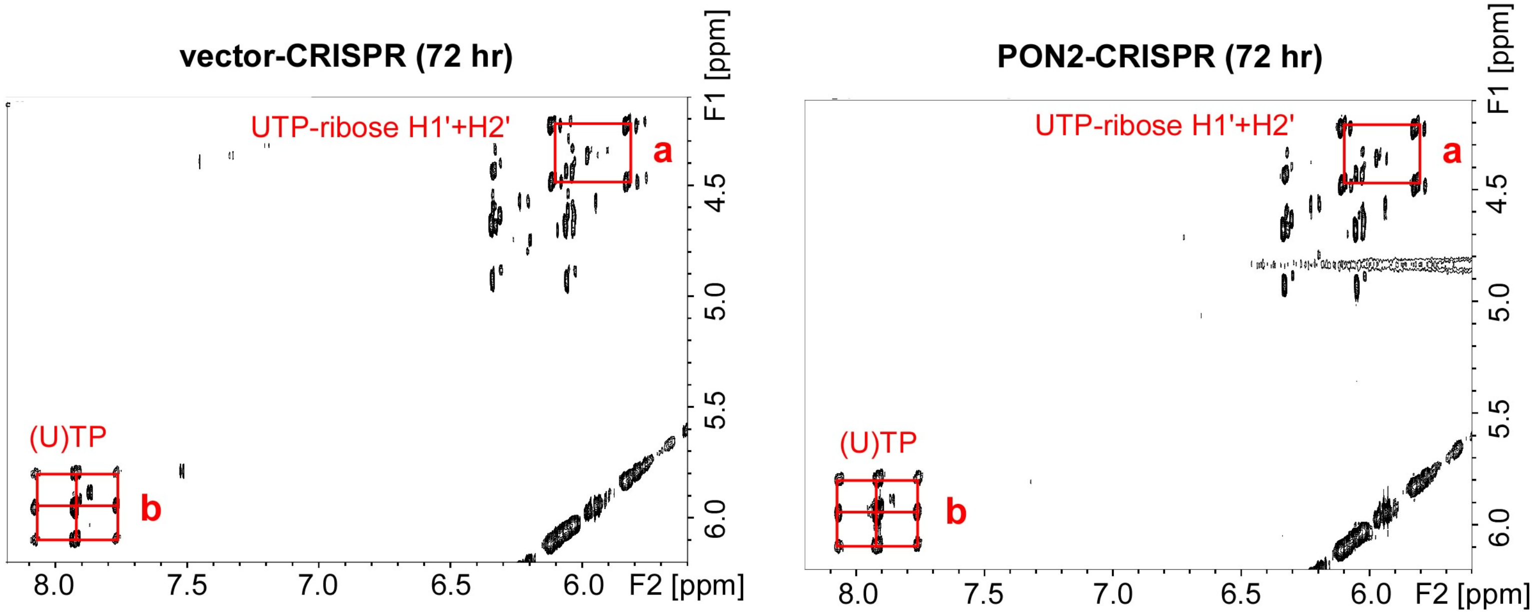

Antibody	Fluorophore	Clone	Manufacturer
CD45	APC/Cy7	30-F11	Biolegend
CD11b	PE/Cy7	M1/70	Biolegend
Gr-1	PE	RB6-8C5	Biolegend
CD38	FITC	90	Biolegend
CD206	BV605	C068C2	Biolegend
F4-80	APC	BM8	eBioscience
Fixable viability dye	Pacific blue	NA	BD Biosciences

Western blot images of Figure 1

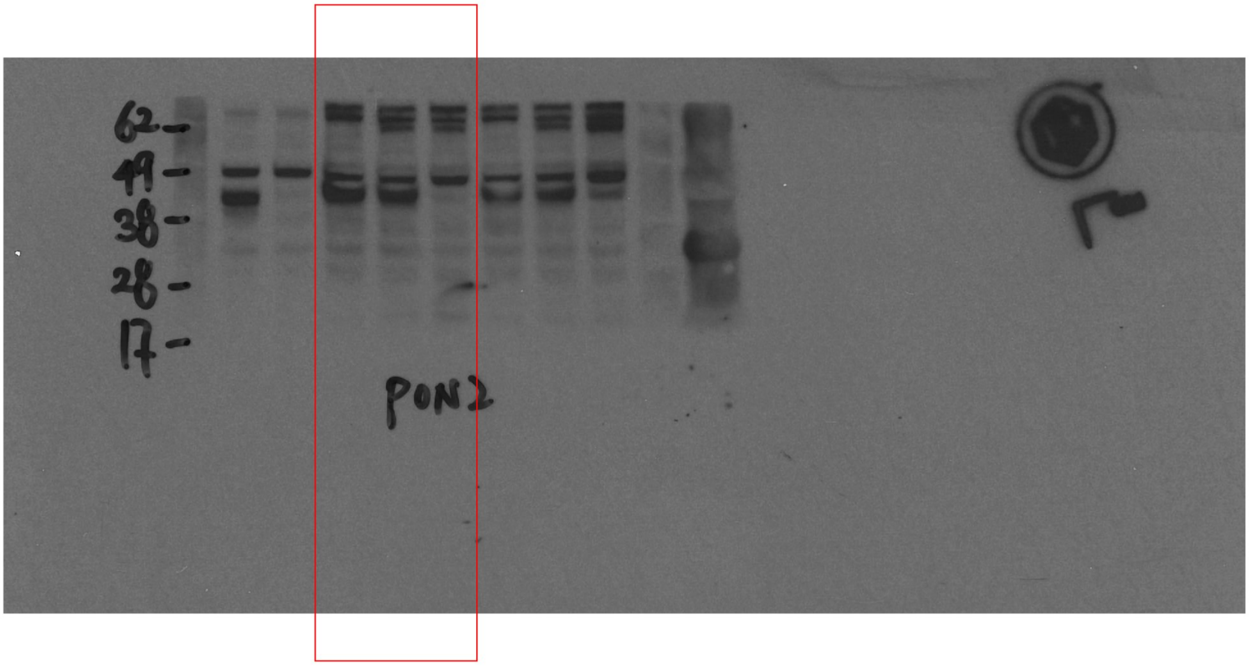

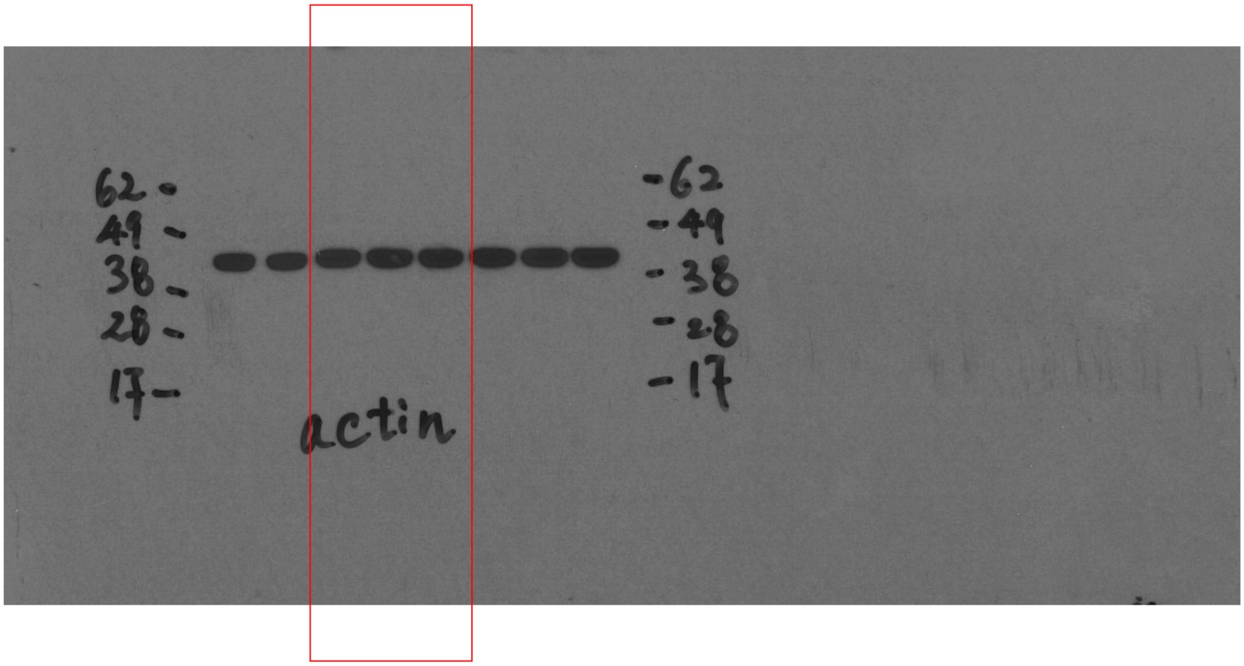

Western blot images of Figure 2A

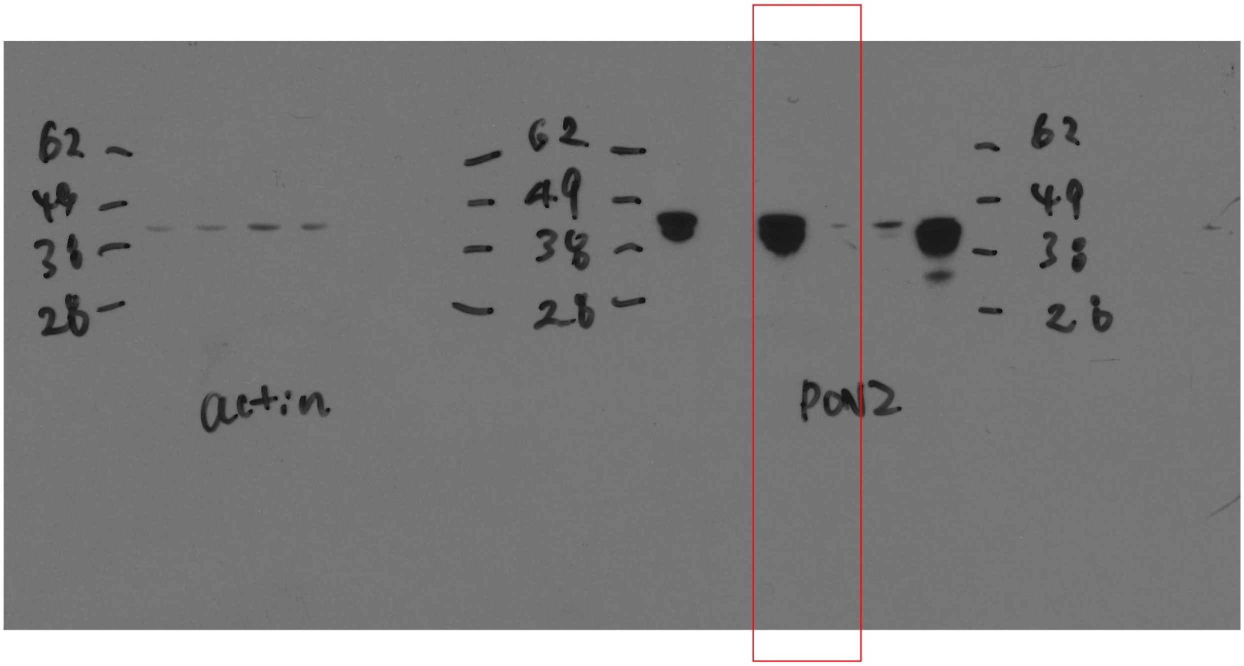

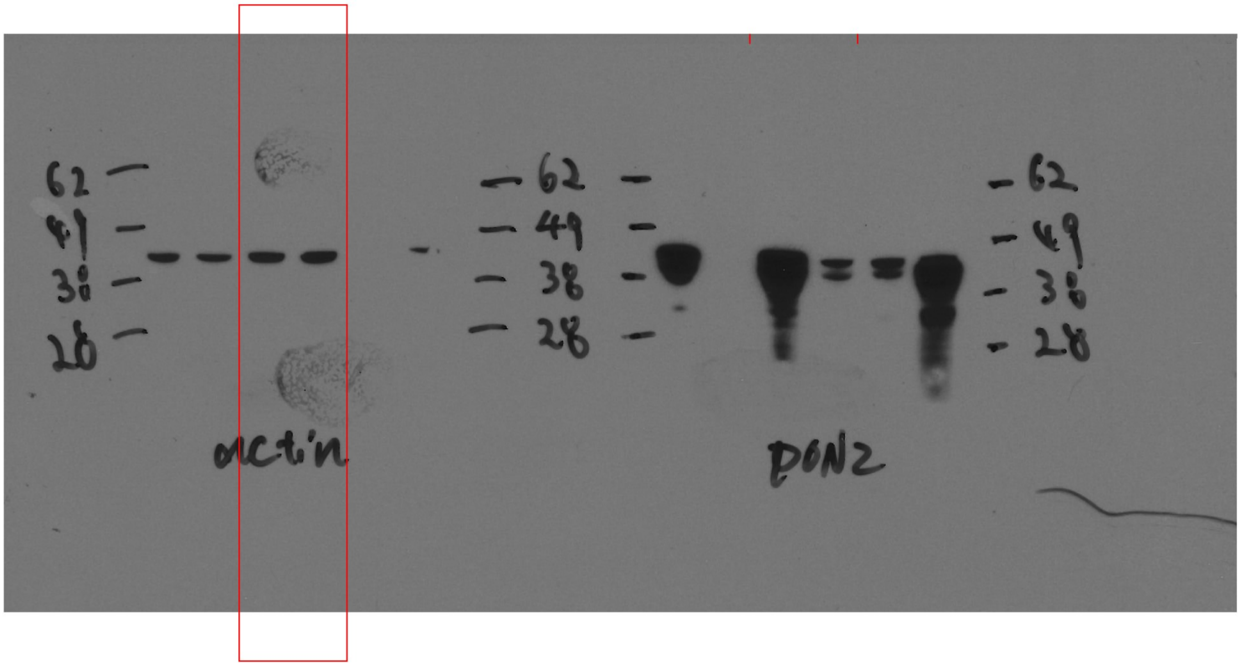

Western blot images of Figure 2E

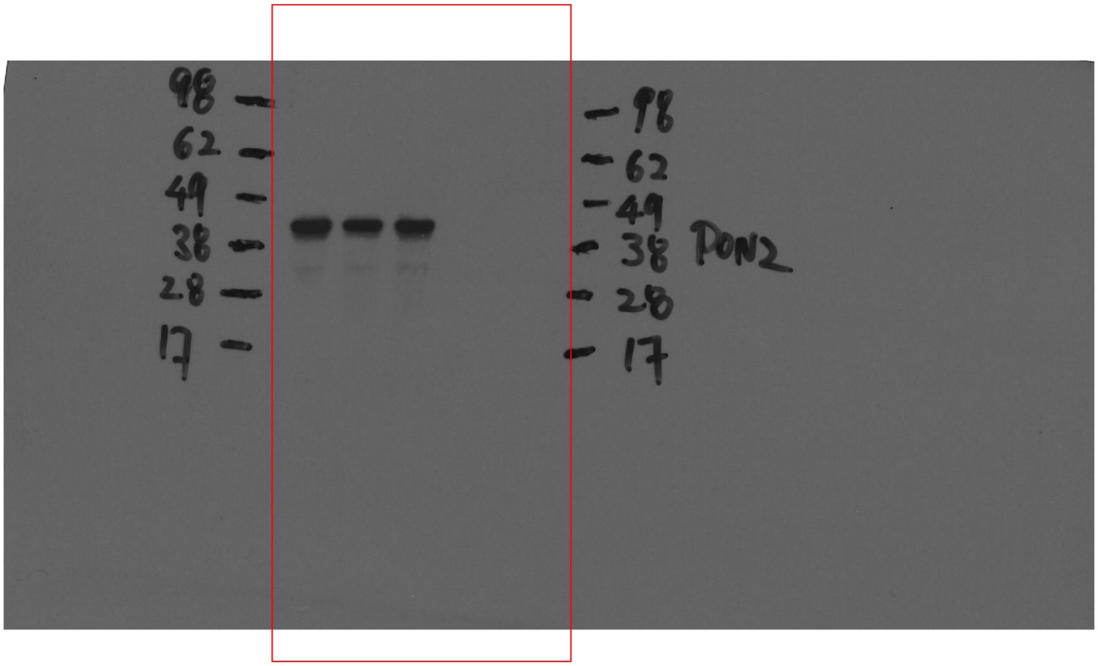

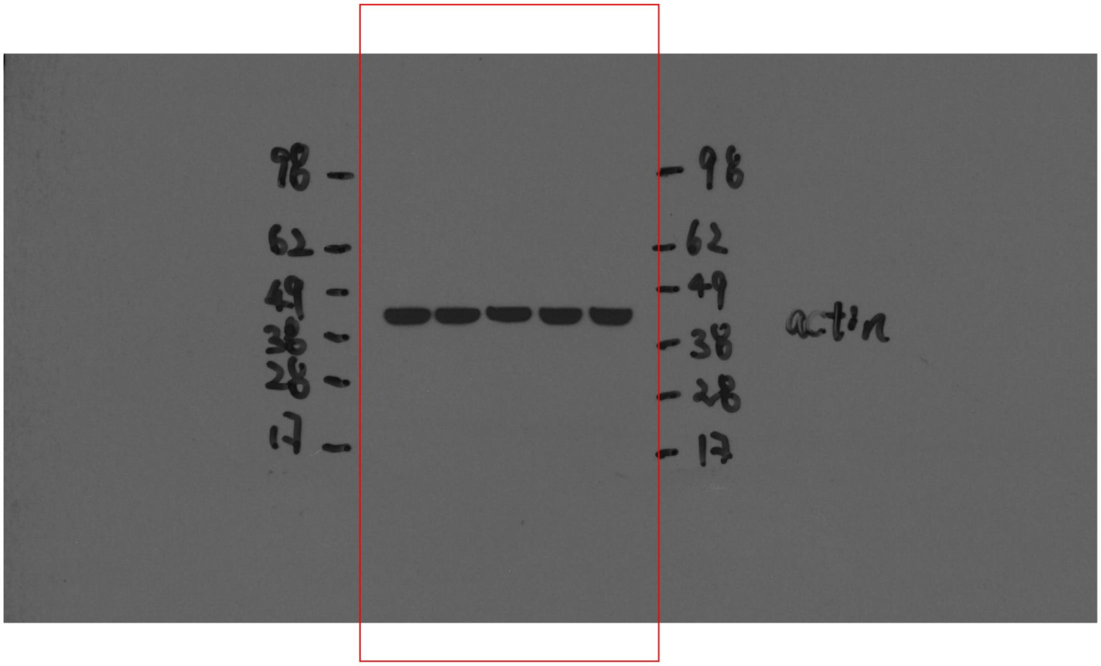

Western blot images of Figure 5A

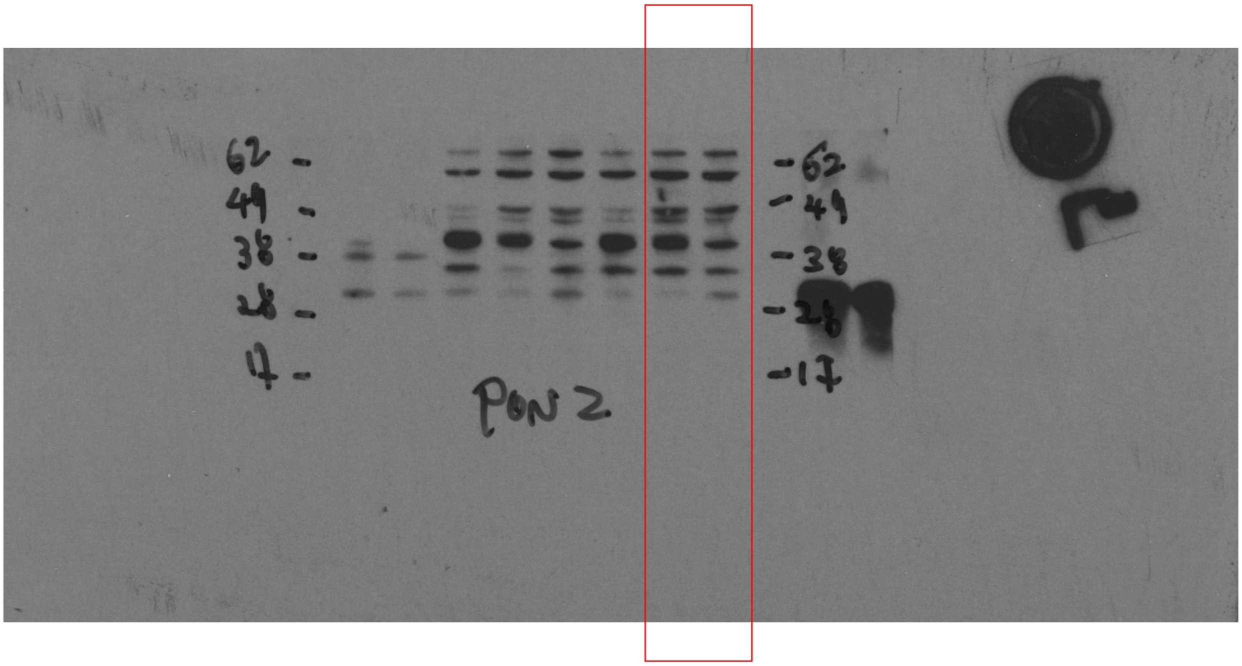

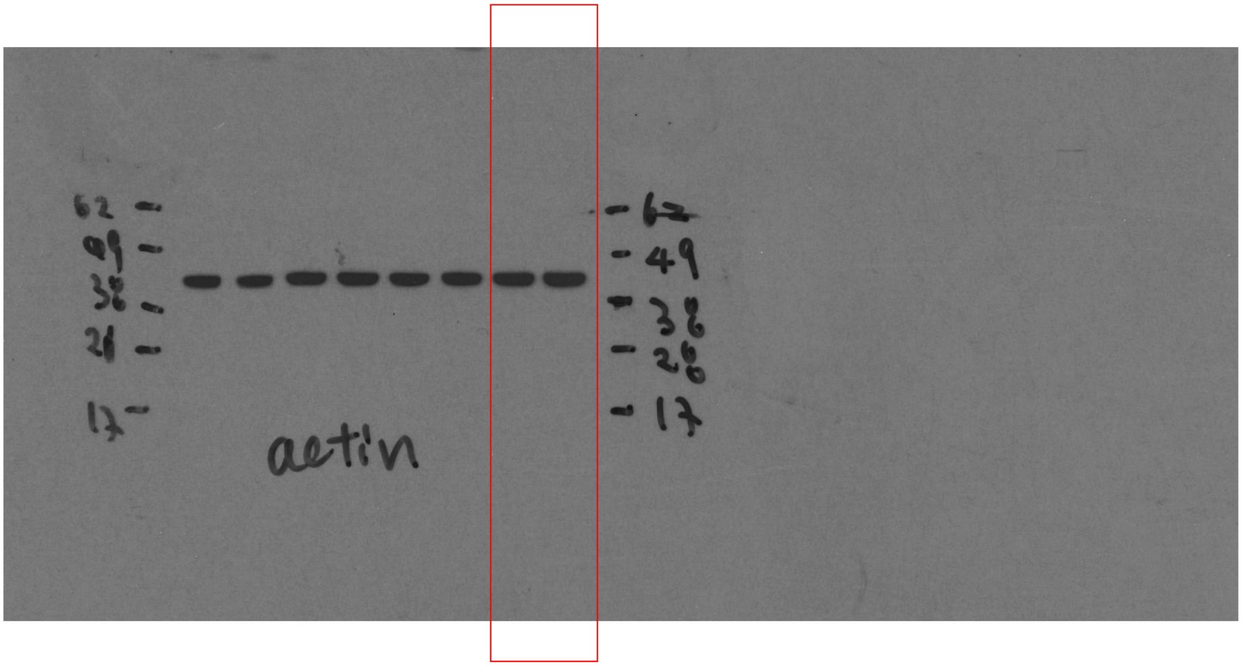

Western blot images of Supplementary figure 1A

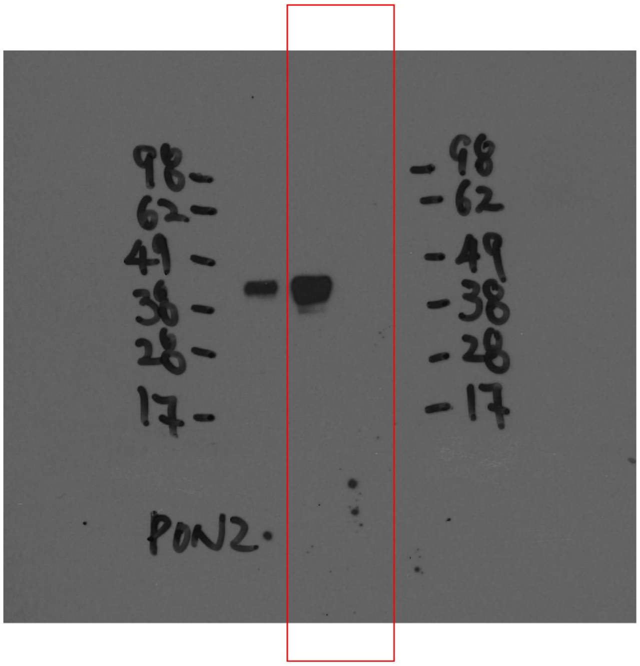

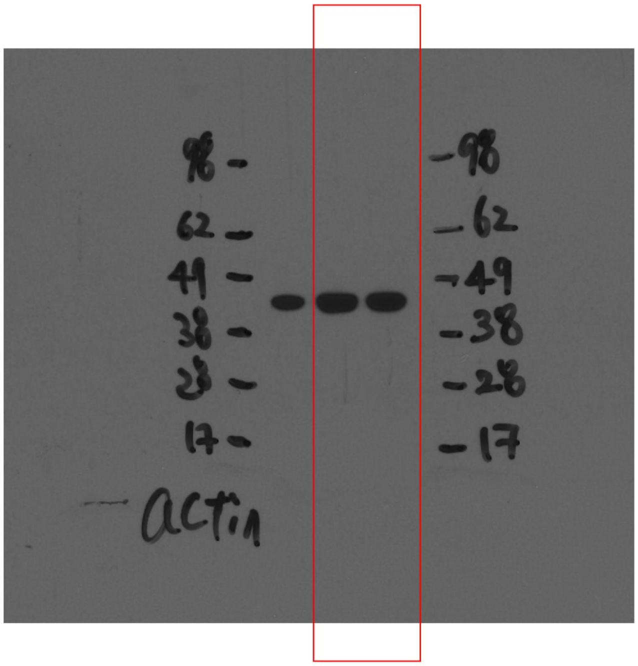

Western blot images of Supplementary figure 1D

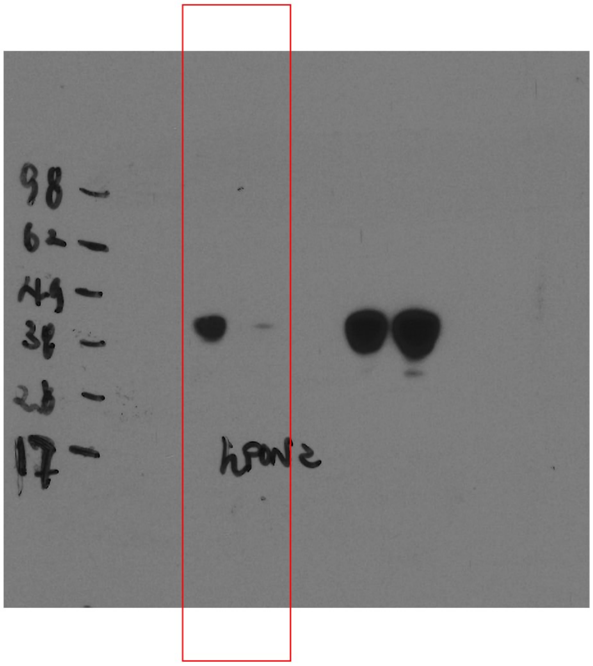

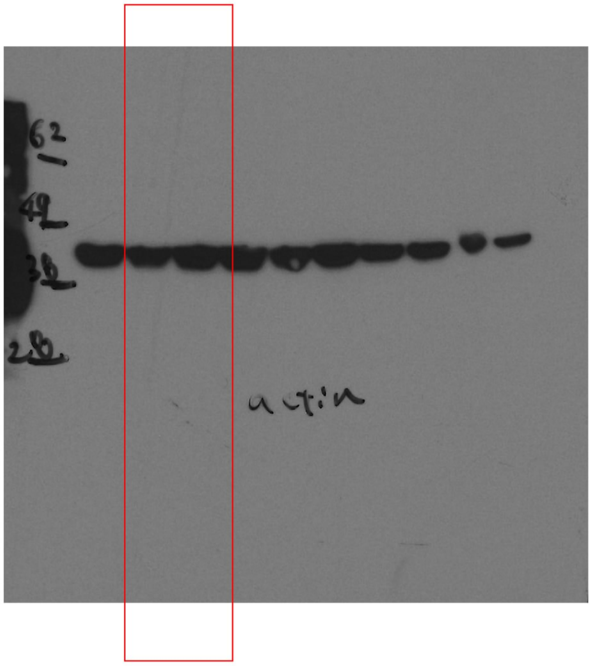

Supplement: Supplementary file 1 — Supplementary Information. [file 41598_2023_37146_MOESM1_ESM.pdf]
